# New Peptide Functionalized Nanostructured Lipid Carriers with CNS Drugs and Evaluation Anti-proliferative Activity

**DOI:** 10.3390/ijms23137109

**Published:** 2022-06-26

**Authors:** Sara Silva, Joana Marto, Lídia M. Gonçalves, Diana Duarte, O. Salomé G. P. Soares, Francisco Vasques-Nóvoa, António J. Almeida, Nuno Vale

**Affiliations:** 1 OncoPharma Research Group, Center for Health Technology and Services Research (CINTESIS), Rua Doutor Plácido da Costa, 4200-450 Porto, Portugal; saracpsilva21@gmail.com (S.S.); dianaduarte29@gmail.com (D.D.); 2 Faculty of Pharmacy, University of Porto, Rua de Jorge Viterbo Ferreira 228, 4050-313 Porto, Portugal; 3 Research Institute for Medicines (iMed.ULisboa), Faculty of Pharmacy, University of Lisbon, 1649-003 Lisbon, Portugal; jmmarto@ff.ulisboa.pt (J.M.); lgoncalves@ff.ulisboa.pt (L.M.G.); aalmeida@ff.ulisboa.pt (A.J.A.); 4 LSRE-LCM-Laboratory of Separation and Reaction Engineering-Laboratory of Catalysis and Materials, Faculty of Engineering, University of Porto, Rua Doutor Roberto Frias, 4200-465 Porto, Portugal; salome.soares@fe.up.pt; 5 ALiCE—Associate Laboratory in Chemical Engineering, Faculty of Engineering, University of Porto, Rua Doutor Roberto Frias, 4200-465 Porto, Portugal; 6 Cardiovascular R&D Center, Faculty of Medicine, University of Porto, Rua Doutor Plácido da Costa, s/n, 4200-450 Porto, Portugal; fvasquesnovoa@med.up.pt; 7 Department of Surgery and Physiology, Faculty of Medicine, University of Porto, 4200-319 Porto, Portugal; 8 Department of Community Medicine, Information and Health Decision Sciences (MEDCIDS), Faculty of Medicine, University of Porto, Rua Doutor Plácido da Costa, s/n, 4200-450 Porto, Portugal; 9 CINTESIS@RISE, Faculty of Medicine, University of Porto, 4200-319 Porto, Portugal

**Keywords:** lipid nanoparticles, cancer cell lines, cell-penetrating peptides, drug repurposing, cytotoxicity assays

## Abstract

Nanoparticulate systems have been widely investigated as delivery vectors for efficient drug delivery in different diseases. Nanostructured lipid carriers (NLC) are composed of both solid and liquid lipids (glyceryl dibehenate and diethylene glycol monoethyl ether) and have demonstrated enhanced biological compatibility and increased drug loading capability. Furthermore, the use of peptides, in particular cell-penetrating peptides, to functionalize nanoparticles and enhance cell membrane permeation was explored in this paper. In this paper, we described the synthesis of a new conjugated of tranylcypromine with MAP. In addition, taking into consideration our previous results, this study developed different NLCs loaded with three central nervous system (CNS) drugs (tacrine (TAC), rasagiline (RAS), and tranylcypromine (TCP)) functionalized with model amphipathic peptide (MAP) and evaluated their activity against cancer cells. Particle size analysis demonstrated NLC presented less than 200 nm and a polydispersity index less than 0.3. Moreover, in vitro results showed that conjugation of MAP with drugs led to a higher decrease in cell viability of a neuroblastoma cell line and Caco-2 cell line, more than MAP alone. Furthermore, NLC encapsulation contributed to higher cellular delivery and enhanced toxic activity at lower concentrations when compared with free or co-administration drug-MAP conjugate.

## 1. Introduction

Cancer has recorded 19.3 million new cases and is responsible for around 10 million deaths worldwide. Breast cancer is considered the most diagnosed cancer followed by lung and colorectal cancer. In addition, lung cancer is associated with the highest mortality rate followed by colorectal and liver cancers [1,2]. Even though current treatment with chemotherapy, surgical removal, and radiotherapy has improved, in some cases leading to patient survival, there are still some cancers for which treatment is limited and ineffective. One example with a poor prognosis is glioblastoma; to the presence of the blood–brain barrier (BBB) limits the amount of chemotherapy and makes it difficult to conduct radiotherapy and some cases surgical approaches [3]. So, new compounds and strategies are required to overcome current treatment limitations. For instance, the development of new drugs for cancer therapy has become a huge challenge, being time-consuming and requiring high investments. For this, a new strategy has been explored to ensure a reduction of costs and a fast-testing track with the use of already-approved drugs with new therapeutic indications other than the original ones. Drug repurposing allows overcoming traditional new anti-cancer drug development limitations and the use of drugs already well characterized in terms of toxicity, side-effects, and dose efficacy [4]. Several compounds already approved have demonstrated some activity against cancer cell lines [5,6]. For instance, studies conducted with TCP demonstrated some anti-cancer activity against different breast cancer cell lines and neuroblastoma cell lines [7,8,9]. However, this anticancer effect was not observed in other cancer cell lines such as human prostate cancer (LNCaP-LN3) cells [10]. TCP was first synthesized in 1948 as an amphetamine analogue. It was later demonstrated as having a potent activity as a non-selective monoamine oxidase (MAO) inhibitor and increased brain catecholamines and serotonin [11,12]. Even though TCP presents a potent anti-depressant activity, the clinical usage was limited to resistant depressions and atypical depressions [13,14] due to its side effects, tyramine-rich food interactions, and drug interactions. Some severe side-effects include hypertensive crisis and central serotonergic syndrome [12,14]. Over the years, several studies have demonstrated that TCP can interact with other types of enzymes such as semicarbazide-sensitive amine oxidases, prostacyclin synthase, drug-metabolizing enzymes, and lysine-specific demethylase 1 (reviewed excellently in ref. [15]). Lysine-specific demethylase 1 overexpression in some cancers has demonstrated to be the possible link of TCP and TCP derivates and harbor anti-proliferation activity against cancer cell lines [16]. 

In a previous work conducted by our group, we demonstrated that conjugation of MAP with TAC resulted in increased toxicity against both MCF-7 and SH-SY5Y cell lines [17]. Moreover, this TAC-MAP conjugated activity was further studied with successful incorporation onto NLC [18]. Even though amphipathic peptides–drug conjugates can possibly form nanoobjects by self-assembly (developed in references [19,20,21]), we loaded them into nanoparticles to ensure increase systemic bioavailability, avoid protein adsorption to the conjugate, and increase efficacy. NLC have been use as a great alternative for solid lipid nanoparticles (SLN) due to the use of liquid lipids in the formulation, which enables the incorporation of higher drug loads and enhances encapsulation efficiency of the overall system [22]. Another great advantage in that these types of nanoparticle systems is that they present decreased toxicity risks due to organic solvent-free preparation upon formulation. In addition, many research groups demonstrated the potential use of these lipid based nanoparticles against several brain diseases [23,24,25,26,27]. Therefore, in this article, we decided to first conjugate TCP with MAP. Then, we explored the use of nanotechnology with NLC to facilitate the delivery of CNS drugs (TAC, RAS and TCP) [28] and drug–MAP conjugates and evaluated their activity against SH-SY5Y human neuroblastoma cell line and colorectal adenocarcinoma Caco-2 cell line. 

## 2. Results

### 2.1. NLC Formulation and Characterization 

The composition of each prepared NLC is described in Table 1. Physicochemical properties of the prepared NLC were analyzed and the overall results are expressed in Table 1. The analysis of all NLC-prepared formulations showed particle size distributions below 160 nm and a polydispersity index below 0.3. The zeta potential analysis demonstrated that all NLC had negative values of −4.13 to −0.38 mV. The EE determined for NLCs loaded with TAC, RAS, and TCP was 35.49%, 63.10%, and 26.02%, respectively. The DL for CT25TAC was 1.57%, CT2RAS was 0.33%, and CT25TCP was 1.57% (Table 1). The data obtained for the peptide encapsulation demonstrated an EE of 12.58%, 18.21%, 14.21%, and 58.34% for CT25 Lys(N_3_)-MAP, CT25TAC-MAP, CT25TCP-MAP, and CT25RAS-MAP, respectively. Furthermore, co-encapsulation of CNS drug and peptide Lys(N_3_)-MAP demonstrated a peptide EE of 2.70% for CT25 Lys(N_3_)-MAP + TAC, 18.70% for CT25 Lys(N_3_)-MAP + TCP, and 35.38% for CT25 Lys(N_3_)-MAP + RAS (see Table 1). For instance, we observed a DL increase with the encapsulation of peptides and peptide–drug conjugates. 

### 2.2. In Vitro Cytotoxicity Assays against Caco-2 Cell Line 

#### 2.2.1. Toxicity of MAP, Lys(N_3_)–MAP and Drug–MAP Conjugates

**Figure 1:**
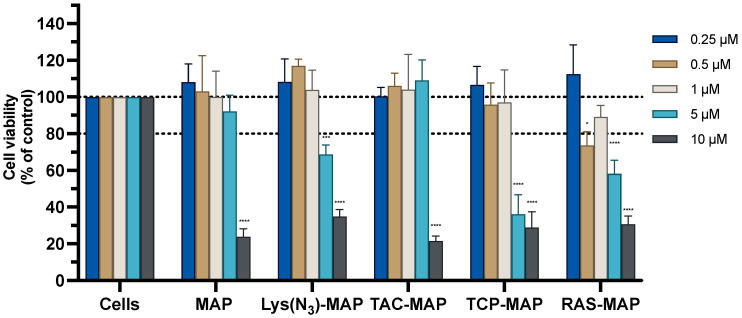
Relative cell viability of the Caco-2 cell line measured by the MTT reduction after 24 h incubation. Treatment with free MAP, Lys(N_3_)-MAP, TAC-MAP, TCP-MAP, and RAS-MAP (0.25–10 µM). Results are expressed as mean ± SD (n = 3 or 6). Statistical analysis between the control groups was performed using two-way ANOVA with Tukey’s multiple comparisons test (* *p* < 0.05; *** *p* < 0.001; **** *p* < 0.0001).

The MTT assay conducted on Caco-2 cells with the treatment of MAP and TAC-MAP demonstrated a significant decrease of cell viability of 20% at 10 µM. On the other hand, Lys(N_3_)-MAP, TCP-MAP, and RAS-MAP demonstrated more toxicity reflected on the decrease of cell viability of 70%, 40%, and 60% at 5 µM, respectively (Figure 1). The SRB results corroborate with MTT analysis even not reflecting the same values of toxicity (Supplementary Figure S10).

**Figure 2:**
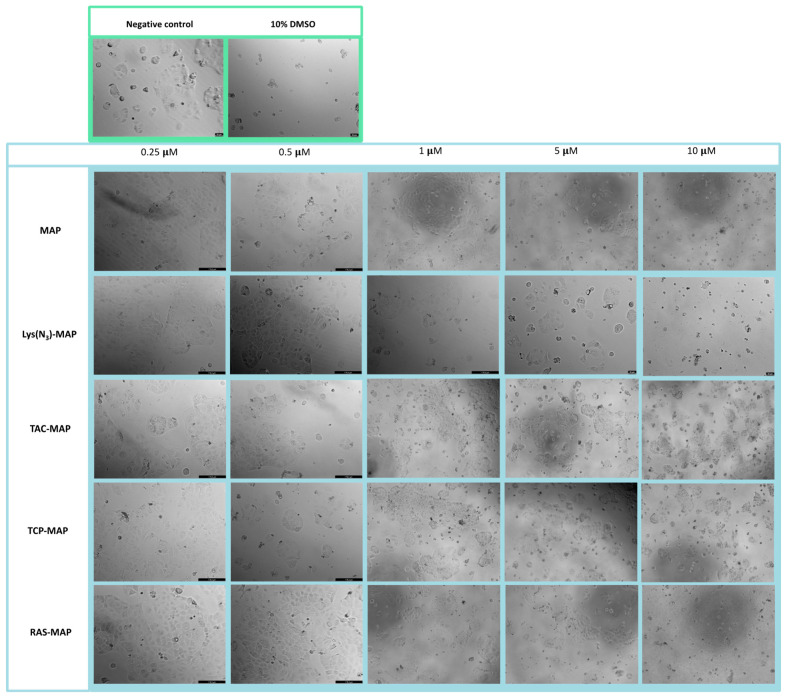
Microscope cellular visualization through Lionheart FX and Leica DFC350 FX of treatment with negative control (ultra-purified H_2_O), positive control (10% DMSO), free peptides, and conjugates on Caco-2 cell line at concentrations of 0.25, 0.5, 1, 5, and 10 μM respectively.

The morphology of the Caco-2 cell line was evaluated after 24 h incubation with all formulations (Figure 2). The analysis conducted through microscope visualization was able to demonstrate and corroborate the results obtain from MTT and SRB assays. As shown in Figure 2, treatment with MAP at 10 µM and Lys(N_3_)-MAP at 5 µM and 10 µM showed a decrease of cell viability and substantial morphologic changes, supporting in vitro cytotoxicity data. On the other hand, the conjugates (TAC-MAP, TCP-MAP, and RAS-MAP) demonstrated higher toxicity at 10 µM with decreased cell viability and a decrease of normal cellular Caco-2 growth in clusters (Figure 2). 

#### 2.2.2. Toxicity of NLC Loaded with TCP, Lys(N_3_)-MAP and TCP-MAP

**Figure 3:**
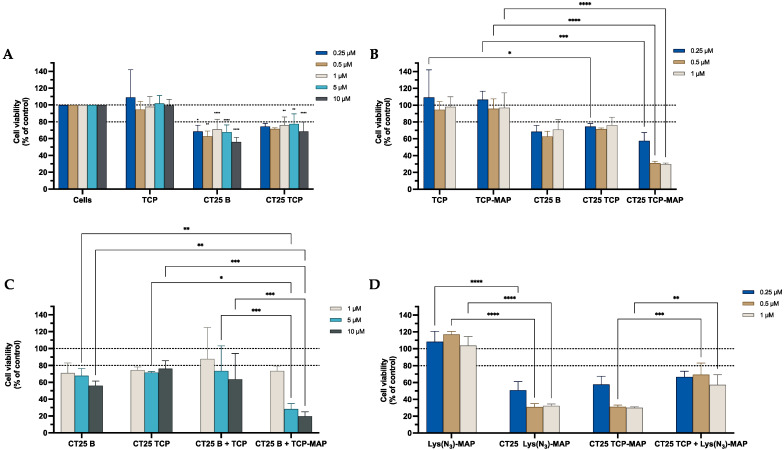
Relative cell viability of Caco-2 cell line measured by the MTT reduction after 24 h incubation. (**A**) represents NLC safety test with the treatment of free TCP, blank CT25, and TCP-loaded CT25 (0.25–10 µM). (**B**) Represents differences in the cell viability of treatment with free TCP, TCP-MAP, blank CT25, TCP-loaded CT25, MAP, and TCP-MAP-loaded CT25 (0.25–10 µM). (**C**) Represents co-administration of TCP and TCP-MAP with blank CT25 (1–10 µM). (**D**) Represents the data obtained from the encapsulation of Lys(N_3_)-MAP and co-encapsulation of TCP and Lys(N_3_)-MAP in the NLC compared with peptide conjugate. Results are expressed as mean ± SD (n = 4). Statistical analysis between the groups was performed using two-way ANOVA with Tukey’s multiple comparisons test (* *p* < 0.05; ** *p* < 0.01; *** *p* < 0.001; **** *p* < 0.0001).

The data from the MTT assay in Figure 3A demonstrated that CT25B and CT25TCP treatment resulted in a decrease of around 20% on cell viability on all concentrations tested. As shown in Figure 3B, the data demonstrated a significant cell viability decrease of 50% to 80% on NLC-loaded TCP-MAP when compared with free TCP-MAP upon administration in the Caco-2 cell line. In addition, co-administration of TCP or TCP-MAP with CT25 Blank NLC did not contribute to enhance of cell toxicity when compared with loaded formulations (Figure 3C). In Figure 3D, we conducted an assay to address co-encapsulation toxicity and the results demonstrated that TCP-MAP promotes significantly more toxicity on the Caco-2 cell line compared with the two compounds TCP and Lys(N_3_)-MAP co-encapsulated on lower concentrations of 0.5 µM and 1 µM and cell viability decreased to around 30% for CT25 TCP-MAP and 50% for CT25 TCP+ Lys(N_3_)-MAP. In the Supplementary Materials, the SRB results corroborate with MTT analysis (Figure S11). 

**Figure 4:**
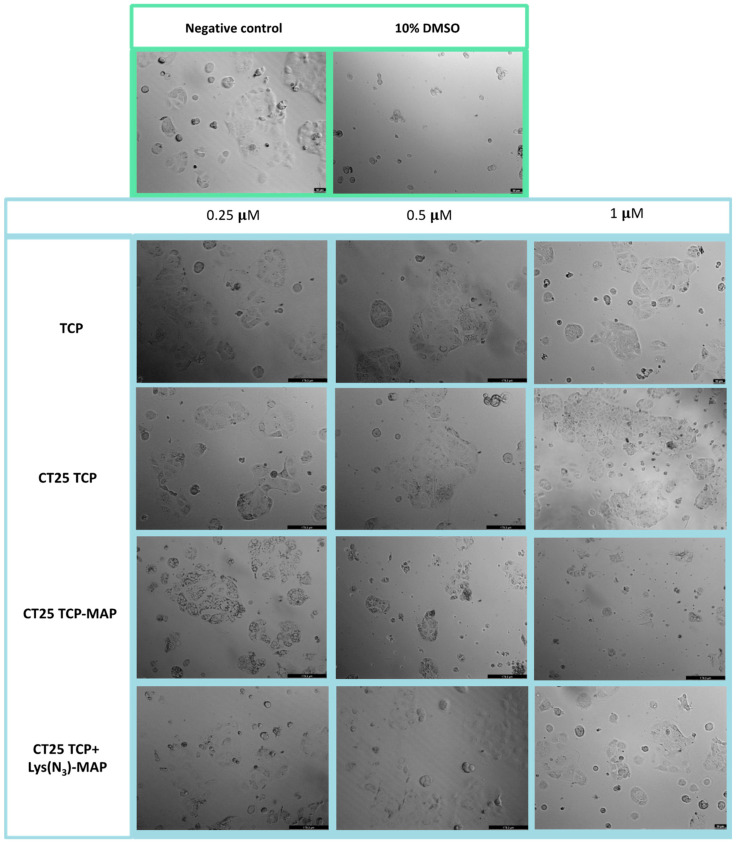
Microscope cellular visualization through Leica DFC350 FX of treatment with negative control (ultra-purified H_2_O), positive control (10% DMSO), free drug, and NLC-loaded formulations (CT25TCP, CT25TCP-MAP and CT25 TCP + Lys(N_3_)-MAP) on Caco-2 cell line at concentrations of 0.25, 0.5, and 1 µM.

Microscope visualization in Figure 4 showed that treatment with CT25TCP did not result in a decrease of cell viability, neither morphological change in Caco-2 cells when compared to the free TCP (Figure 4—TCP 0.25, 0.5, and 1 µM). Co-administration of TCP and TCP-MAP with CT25 blank (CT25 TCP + MAP and CT 25 B + TCP-MAP) microscope images showed corroboration with MTT assay (Figure S15). On the other hand, as the MTT data, CT25TCP-MAP treatment resulted in a decrease of cell viability and severe morphological changes represented in Figure 4 at all concentrations tested; only at 0.25 µM did we observe cytoplasmatic granulation inside the Caco-2. Once again, co-encapsulation of the TCP and Lys(N_3_)-MAP (CT25 TCP + Lys(N_3_)-MAP) showed a decrease of cell viability but no morphological changes at 1 µM concentration. 

#### 2.2.3. Toxicity of NLC Loaded with RAS, Lys(N_3_)-MAP and RAS-MAP

**Figure 5:**
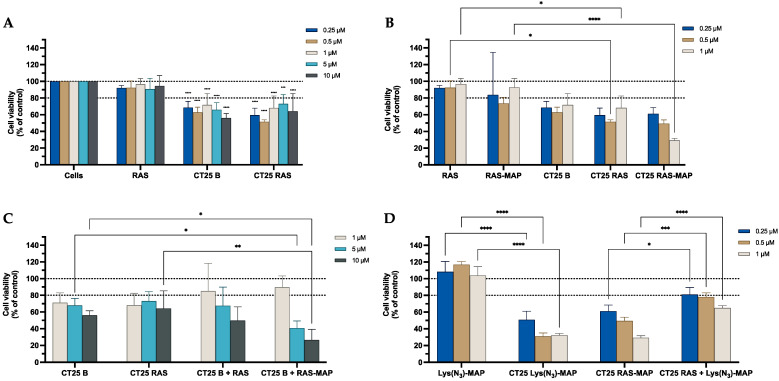
Relative cell viability of Caco-2 cell line measured by the MTT reduction after 24 h incubation. (**A**) Represents NLC safety test with the treatment of free RAS, blank CT25 and RAS-loaded CT25 (0.25–10 µM). (**B**) Represents differences in the cell viability of treatment with free RAS, RAS-MAP, blank CT25, RAS-loaded CT25, MAP, RAS-MAP-loaded CT25 (0.25–10 µM). (**C**) Represents co-administration of RAS and RAS-MAP with blank CT25 (1–10 µM). (**D**) Represents the data obtained from the encapsulation of Lys(N_3_)-MAP and co-encapsulation of RAS and Lys(N_3_)-MAP in the NLC compared with peptide conjugate. Results are expressed as mean ± SD (n = 4 or 6). Statistical analysis between the groups was performed using two-way ANOVA with Tukey’s multiple comparisons test (* *p* < 0.05; ** *p* < 0.01; *** *p* < 0.001; **** *p* < 0.0001).

The results obtained from the MTT assay, shown in Figure 5A, showed that CT25B and CT25RAS treatment resulted in a decrease of around 20% on cell viability on all concentrations tested. The cell viability showed a significant decrease to 30% on NLC-loaded RAS-MAP when compared with free RAS-MAP at the concentration of 1 µM (Figure 5B). Furthermore, co-administration of RAS or RAS-MAP with CT25 Blank NLC did not contribute to a significant enhancement of cell toxicity when compared to loaded formulations (Figure 5C). The co-encapsulation of RAS and Lys(N_3_)-MAP in the NLC showed that RAS-MAP promotes significantly more toxicity on the Caco-2 cell line when compared with the two compounds RAS and Lys(N_3_)-MAP co-encapsulated at all studied concentrations. Cell viability decreased to around 60%, 50%, and 30% on CT25 RAS-MAP and 80%, 78%, and 65% for CT25 TCP+ Lys(N_3_)-MAP at 0.25 µM, 0.5 µM, and 1 µM, respectively (Figure 5D). In the Supplementary Materials, the SRB results corroborate with MTT analysis (Figure S12).

**Figure 6:**
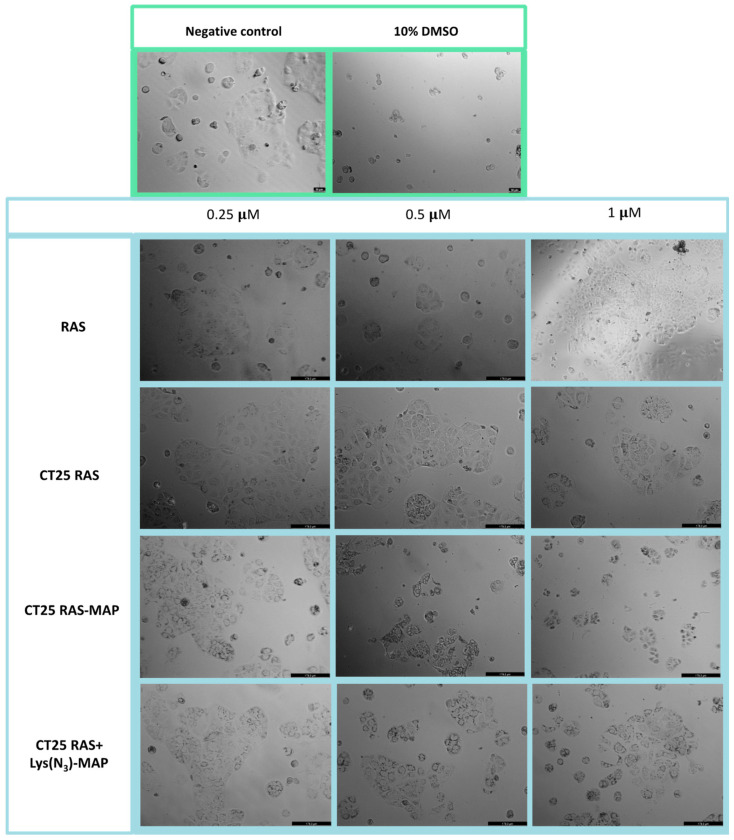
Microscope cellular visualization through Leica DFC350 FX of treatment with negative control (ultra-purified H_2_O), positive control (10% DMSO), free drug, and NLC-loaded formulations ((CT25RAS, CT25RAS-MAP and CT25 RAS + Lys(N_3_)-MAP) on Caco-2 cell line at concentrations of 0.25, 0.5, and 1 µM.

The morphological evaluation of the Caco-2 cell line in Figure 6 showed that treatment with CT25RAS did not result in a decrease in cell viability nor morphological change when compared to the free TCP and negative control (Figure 6). Co-administration of RAS and RAS-MAP with CT25 blank microscope images showed corroboration with MTT assay (Figure S15). With the data obtained from the MTT assay, CT2RAS-MAP treatment showed a decrease of cell viability and severe morphological changes represented in Figure 6 at all concentrations tested. Once again, co-encapsulation of the RAS and Lys(N_3_)-MAP (CT25 RAS + Lys(N_3_)-MAP) showed less decrease in cell viability, but we could observe some morphological changes at 0.5 and 1 µM concentrations with cytoplasmatic granulation. 

#### 2.2.4. Toxicity of NLC Loaded with TAC, Lys(N_3_)-MAP and TAC-MAP

**Figure 7:**
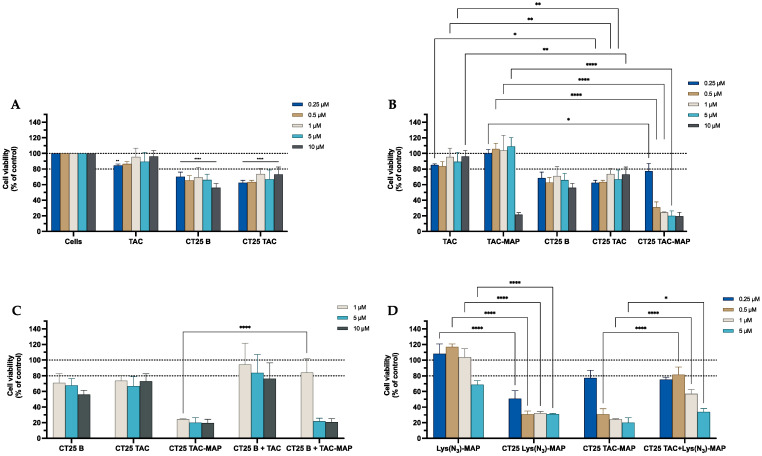
Relative cell viability of Caco-2 cell line measured by the MTT reduction after 24h incubation. (**A**) represents NLC safety test with the treatment of free TAC, blank CT25 and TAC-loaded CT25 (0.25–10 µM). (**B**) Represents differences in the cell viability of treatment with free TAC, TAC-MAP, blank CT25, TAC-loaded CT25, MAP, TAC-MAP-loaded CT25 (0.25–10 µM). (**C**) Represents co-administration of TAC and TAC-MAP with blank CT25 (1–10 µM). (**D**) Represents the data obtained from the encapsulation of Lys(N_3_)-MAP and co-encapsulation of TAC and Lys(N_3_)-MAP in the NLC compared with peptide conjugate. Results are expressed as mean ± SD (n = 4). Statistical analysis between the groups was performed using two-way ANOVA with Tukey’s multiple comparisons test (* *p* < 0.05; ** *p* < 0.01; **** *p* < 0.0001).

The MTT assays shown in Figure 7A demonstrate that CT25B and CT25TAC treatment resulted in a significant decrease of around 30% on cell viability at all concentrations tested. For instance, in Figure 7B the data demonstrated a significant cell viability decrease on NLC-loaded TAC-MAP when compared with free TAC-MAP on all concentrations tested. Moreover, no differences were observed on co-administration of TAC or TAC-MAP with CT25 Blank NLC test when compared to loaded formulations (Figure 7C). As shown in Figure 7D, we analyzed the difference of co-encapsulation toxicity on the Caco-2 cell line and the results demonstrated that CT25TAC-MAP promotes significant more toxicity when compared with the two compounds TAC and Lys(N_3_)-MAP co-encapsulated at concentrations of 0.5, 1, and 5 µM with and cell viability decreases to around 30% CT25 TAC-MAP. The SRB results corroborate with MTT analysis (Figure S13).

**Figure 8:**
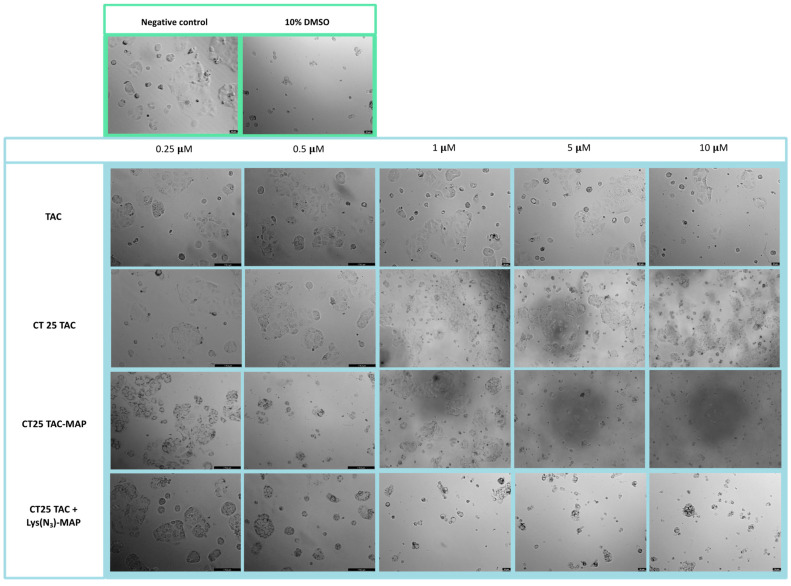
Microscope cellular visualization through Lionheart FX and Leica DFC350 FX of treatment with negative control (ultra-purified H_2_O), positive control (10% DMSO), free drug, and NLC-loaded formulations (CT25TAC, CT25TAC-MAP and CT25 TAC + Lys(N_3_)-MAP) on Caco-2 cell line at concentrations of 0.25, 0.5, 1, 5, and 10 μM.

Microscope visualization of the treatment with CT25TAC showed no apparent decrease in cell viability and no morphological changes at all concentrations tested when compared to negative control and free TAC (Figure 8). The co-administration test of TAC and TAC-MAP with CT25 blank microscope (CT25 TAC + MAP and CT 25 B + TAC-MAP) images showed corroboration with MTT assay with a decrease in cell viability and severe morphological changes on cell structure (Figure S15). The microscope images obtained after CT2TAC-MAP treatment showed a decrease in cell viability and severe morphological changes, as represented in Figure 8 in the concentration of 0.5 µM. In this case, co-encapsulation of the TAC and Lys(N_3_)-MAP (CT25 TAC + Lys(N_3_)-MAP) showed a decrease in cell viability and observed morphological changes at of 1, 5, and 10 µM concentration with complete loss of cell structure and decrease cellular size. 

### 2.3. In Vitro Cytotoxicity Assays against SH-SY5Y

#### 2.3.1. Toxicity of MAP, Lys(N_3_)–MAP and Drug–MAP Conjugates

**Figure 9:**
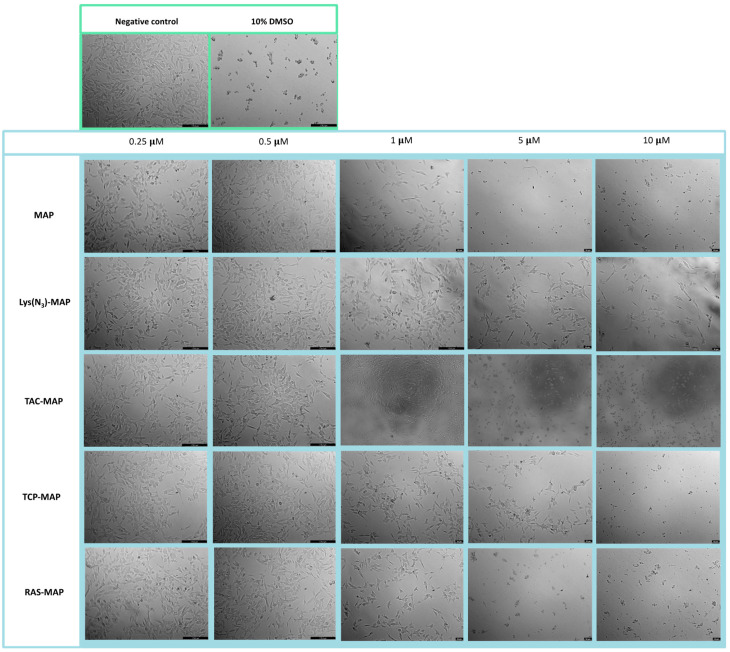
Microscope cellular visualization through Lionheart FX and Leica DFC350 FX of treatment with negative control (ultra-purified H_2_O), positive control (10% DMSO), free peptides, and conjugates on SH-SY5Y cell line at concentrations of 0.25, 0.5, 1, 5, and 10 μM.

**Figure 10:**
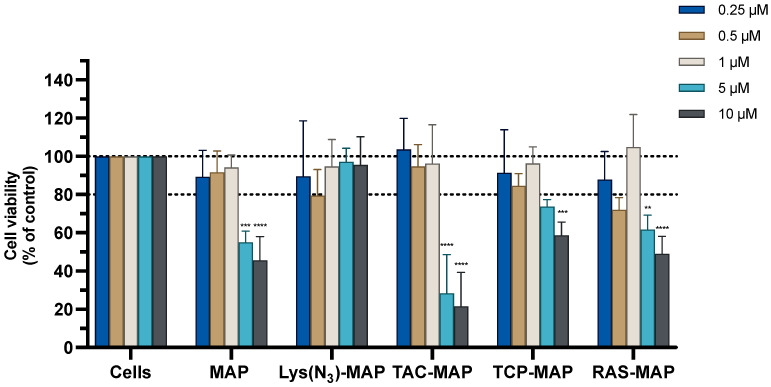
Relative cell viability of SH-SY5Y cell line measured by the MTT reduction after 24 h incubation. Treatment with free MAP, Lys(N_3_)-MAP, TAC-MAP, TCP-MAPm and RAS-MAP (0.25–10 µM). Results are expressed as means ± SD (n = 3 or 6). Statistical analysis between the control groups was performed using two-way ANOVA with Tukey’s multiple comparisons test (** *p* < 0.01; *** *p* < 0.001; **** *p* < 0.0001).

The morphology of SH-SY5Y was evaluated after 24 h incubation with all formulations (Figure 9). The analysis conducted through microscope visualization showed that treatment with MAP at 1 µM and Lys(N_3_)-MAP at 5 µM decreased substantial cell viability and showed some morphologic change with loss of normal cellular structure and round aspect. Moreover, treatment with free TCP-MAP, RAS-MAP, and TAC-MAP resulted in a decrease of cell density at 1 µM and severe morphological changes with loss of cell structure at 5 µM and 10 µM (Figure 10). 

#### 2.3.2. Toxicity of NLC Loaded with TCP, Lys(N_3_)-MAP and TCP-MAP

**Figure 11:**
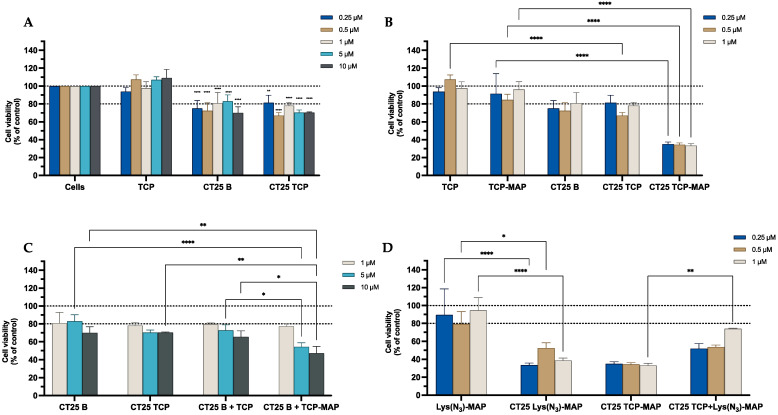
Relative cell viability of SH-SY5Y cell line measured by the MTT reduction after 24 h incubation. (**A**) represents NLC safety test with the treatment of free TCP, blank CT25 and TCP-loaded CT25 (0.25–10 µM). (**B**) Represents differences in the cell viability of treatment with free TCP, TCP-MAP, blank CT25, TCP-loaded CT25, MAP, TCP-MAP-loaded CT25 (0.25–10 µM). (**C**) Represents co-administration of TCP and TCP-MAP with blank CT25 (1–10 µM). (**D**) Represents the data obtained from the encapsulation of Lys(N_3_)-MAP and co-encapsulation of TCP and Lys(N_3_)-MAP in the NLC compared to peptide conjugate. Results are expressed as means ± SD (n = 4). Statistical analysis between the groups was performed using two-way ANOVA with Tukey’s multiple comparisons test (* *p* < 0.05; ** *p* < 0.01; **** *p* < 0.0001).

The data from the MTT assay shown in Figure 11A show that CT25B and CT25TCP treatment resulted in a decrease of around 25% on cell viability on all concentrations tested against neuroblastoma cell line SH-SY5Y. As shown in Figure 11B, the data demonstrated a significant cell viability decrease of around 70% on NLC-loaded TCP-MAP when compared with free TCP-MAP. In addition, co-administration of TCP or TCP-MAP with CT25 Blank NLC lead to similar cell toxicity profiles when compared to loaded formulations and free compound administration (Figure 11C). Moreover, results shown in Figure 11D demonstrate that co-encapsulation of TCP and Lys(N_3_)-MAP did not promote significant toxicity on the SH-SY5Y cell line when compared to the CT25TCP-MAP on all concentrations tested. The results demonstrated a cell viability decrease to around 30% on CT25 TCP-MAP treatment and to around 65% for CT25 TCP+ Lys(N_3_)-MAP. The SRB results showed the same toxic profile of the MTT analysis (Figure S17).

**Figure 12:**
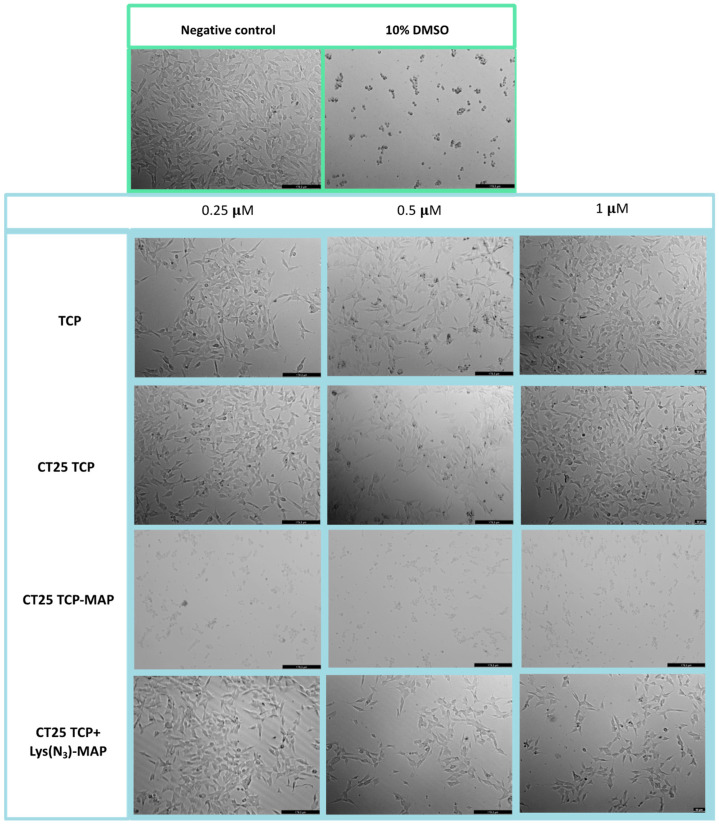
Microscope cellular visualization through Leica DFC350 FX of treatment with negative control (ultra-purified H_2_O), positive control (10% DMSO), free drug, and NLC-loaded formulations (CT25TCP, CT25TCP-MAP and CT25 TCP + Lys(N_3_)-MAP) on SH-SY5Y cell line at a concentration of 0.25, 0.5, and 1 µM.

Microscope visualization showed that treatment with CT25TCP showed no alteration on cell viability and no morphological changes on SH-SY5Y when compared to negative control and free TCP (Figure 12). MTT assay showed no differences on co-administration of TCP and TCP-MAP with CT25 blank (CT25 TCP + MAP and CT 25 B + TCP-MAP) and the microscope images showed similar results with decreased cell viability at all concentrations tested (Figure S21). Furthermore, CT25TCP-MAP treatment showed a high decrease in cell viability and severe morphological changes with loss of cell structure at all concentrations represented in Figure 12. The co-encapsulation treatment of the TCP and Lys(N_3_)-MAP (CT25 TCP + Lys(N_3_)-MAP) showed a decrease in cell viability at 0.5 µM but no morphological changes at all concentrations (Figure 12). 

#### 2.3.3. Toxicity of NLC Loaded with RAS, Lys(N_3_)-MAP and RAS-MAP

**Figure 13:**
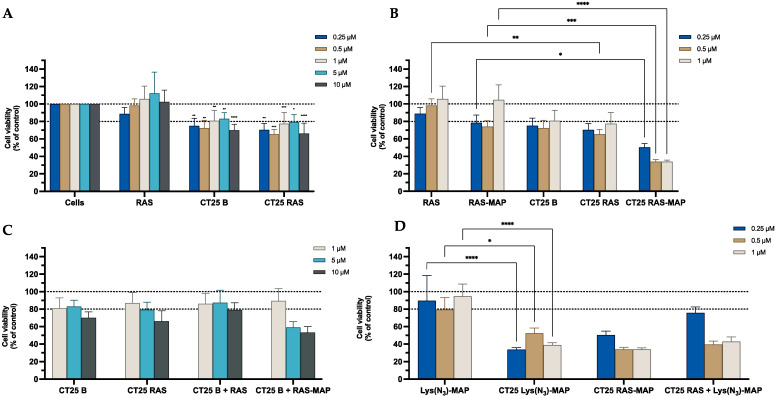
Relative cell viability of SH-SY5Y cell line measured by the MTT reduction after 24 h incubation. (**A**) Represents NLC safety test with the treatment of free RAS, blank CT25 and RAS-loaded CT25 (0.25–10 µM). (**B**) Represents differences in the cell viability of treatment with free RAS, RAS-MAP, blank CT25, RAS-loaded CT25, MAP, RAS-MAP-loaded CT25 (0.25–10 µM). (**C**) Represents co-administration of RAS and RAS-MAP with blank CT25 (1–10 µM). (**D**) Represents the data obtained from the encapsulation of Lys(N_3_)-MAP and co-encapsulation of RAS and Lys(N_3_)-MAP in the NLC compared with peptide conjugate. Results are expressed as means ± SD (n = 4). Statistical analysis between the groups was performed using two-way ANOVA with Tukey’s multiple comparisons test (* *p* < 0.05; ** *p* < 0.01; *** *p* < 0.001; **** *p* < 0.0001).

The results obtained from the MTT assay shown in Figure 13A demonstrate that CT25B and CT25RAS treatment resulted in a decrease of around 25% on cell viability on all concentrations tested. The cell viability showed a significant decrease on NLC-loaded RAS-MAP treatments when compared with free RAS-MAP at all concentrations (Figure 13B). Furthermore, co-administration of RAS or RAS-MAP with CT25 Blank NLC did not contribute to a significant decrease in cell viability when compared to loaded formulations (Figure 13C). Figure 13D showed that CT25RAS-MAP promote slightly more toxicity on the SH-SY5Y cell line when compared with the two compounds RAS and Lys(N_3_)-MAP co-encapsulated at all concentrations studied. Cell viability decreased to around 50%, 30%, and 30% on CT25 RAS-MAP and 75%, 40%, and 45% for CT25 TCP+ Lys(N_3_)-MAP at 0.25 µM, 0.5 µM, and 1 µM, respectively (Figure 13D). The SRB results corroborate with MTT analysis conducted (Figure S18).

**Figure 14:**
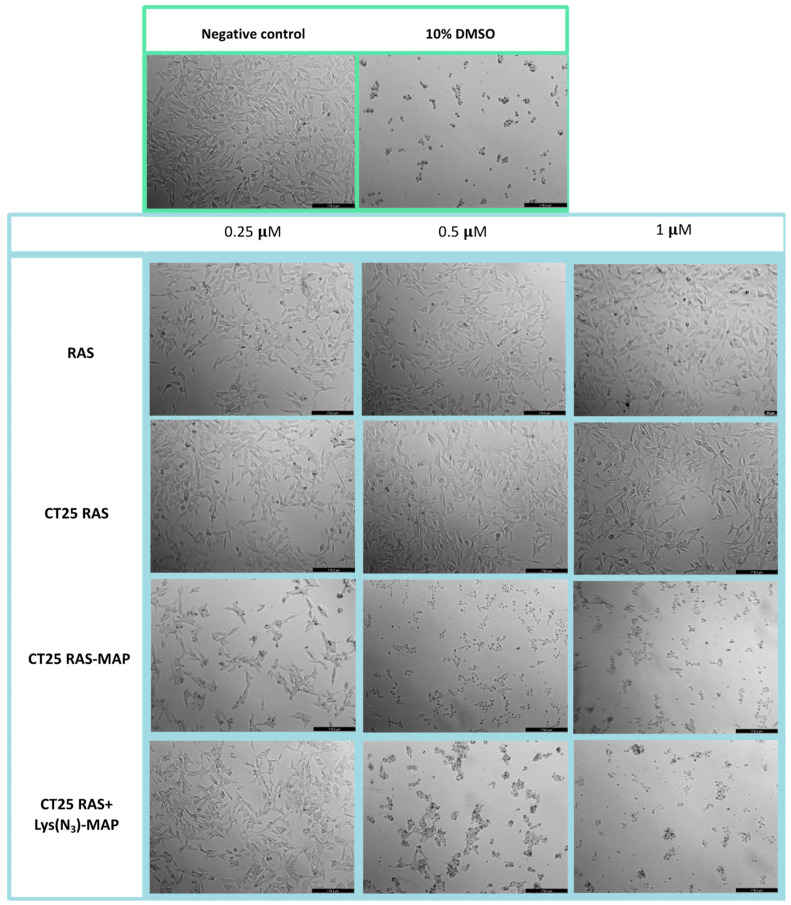
Microscope cellular visualization through Leica DFC350 FX of treatment with negative control (ultra-purified H_2_O), positive control (10% DMSO), free drug, and NLC-loaded formulations (CT25RAS, CT25RAS-MAP and CT25 RAS + Lys(N_3_)-MAP) on SH-SY5Y cell line at a concentration of 0.25, 0.5, and 1 µM.

The morphological evaluation of the neuroblastoma SH-SY5Y cell line showed that treatment with CT25RAS resulted in similar cell viability and no morphologic changes compared to the negative control and free RAS (Figure 14). Co-administration of RAS and RAS-MAP with CT25 blank (CT25 RAS + MAP and CT 25 B + RAS-MAP) microscope images showed corroboration with MTT assay with decreased cellular viability at 5 µM (Figure S21). As shown in Figure 14, the treatment of CT25RAS-MAP showed a severe decrease in cell viability and loss of normal cellular structure at 0.5 µM. The co-encapsulation of the RAS and Lys(N_3_)-MAP (CT25 RAS + Lys(N_3_)-MAP) treatment showed a decrease in cell viability, but we only observed some morphological changes at the concentration of 1 µM (Figure 14).

#### 2.3.4. Toxicity of NLC Loaded with TAC, Lys(N_3_)-MAP and TAC-MAP

**Figure 15:**
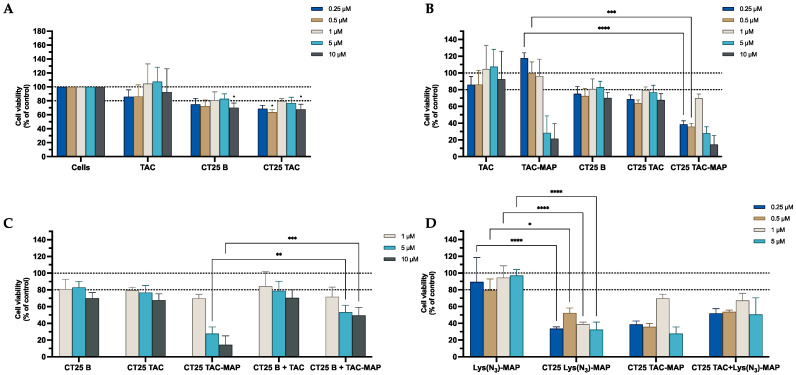
Relative cell viability of SH-SY5Y cell line measured by the MTT reduction after 24 h incubation. (**A**) represents NLC safety test with the treatment of free TAC, blank CT25, and TAC-loaded CT25 (0.25–10 µM). (**B**) Represents differences in the cell viability of treatment with free TAC, TAC-MAP, blank CT25, TAC-loaded CT25, MAP, TAC-MAP-loaded CT25 (0.25–10 µM). (**C**) Represents co-administration of TAC and TAC-MAP with blank CT25 (1–10 µM). (**D**) Represents the data obtained from the encapsulation of Lys(N_3_)-MAP and co-encapsulation of TAC and Lys(N_3_)-MAP in the NLC compared with peptide conjugate. Results are expressed as means ± SD (n = 4). Statistical analysis between the groups was performed using two-way ANOVA with Tukey’s multiple comparisons test (* *p* < 0.05; ** *p* < 0.01; *** *p* < 0.001; **** *p* < 0.0001).

The MTT assays performed on the SH-SY5Y cell line shown in Figure 15A demonstrated that CT25B and CT25TAC treatments resulted in a significant decrease of around 28% on cell viability on all concentrations tested (Figure 15A). For instance, in Figure 15B the data demonstrate a significant cell viability decrease on NLC-loaded TAC-MAP when compared with free TAC-MAP, more pronounced on lower concentrations of 0.25 µM and 0.5 µM. Furthermore, no differences were observed on co-administration of TAC or TAC-MAP with CT25 Blank NLC test when compared to loaded formulations CT25TAC and CT25TAC-MAP (Figure 15C). As shown in Figure 15D, we analyzed the difference of co-encapsulation toxicity and the results demonstrated that CT25TAC-MAP promoted more toxicity than the two compounds TAC and Lys(N_3_)-MAP co-encapsulated at all concentrations. The SRB results corroborate with MTT analysis (Figure S19).

**Figure 16:**
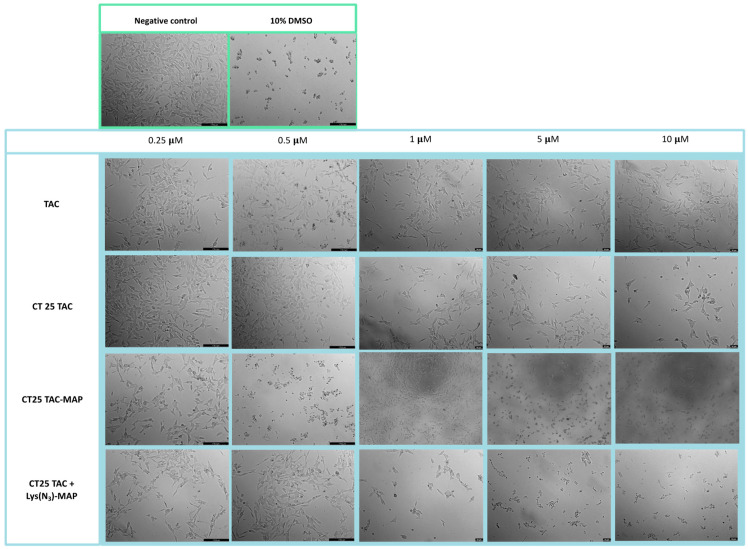
Microscope cellular visualization through Lionheart FX and Leica DFC350 FX of treatment with negative control (H_2_O), positive control (10% DMSO), free drug and NLC-loaded formulations (CT25TAC, CT25TAC-MAP and CT25 TAC + Lys(N_3_)-MAP) on SH-SY5Y cell line at a concentration of 0.25, 0.5, 1, 5 and 10 μM.

Microscope visualization of the treatment with CT25TAC showed a decrease in cell viability and no morphological changes at 1, 5, and 10 µM when compared to negative control and free TAC (Figure 16). The co-administration assay of TAC and TAC-MAP with CT25 blank (CT25 TAC + MAP and CT 25 B + TAC-MAP) microscope images showed corroboration with MTT assay with a decrease in cell viability at 1 µM and severe morphological changes on cell structure at 5 µM and 10 µM (Figure S21). The microscope images obtained with CT2TAC-MAP treatment showed a decrease of cell viability and severe morphological changes represented in Figure 16 in 0.5, 5, and 10 µM. Moreover, co-encapsulation of the TAC and Lys(N_3_)-MAP (CT25 TAC + Lys(N_3_)-MAP) shown in Figure 16 demonstrated a decrease in cell viability and observed morphological changes at of 1, 5, and 10 µM concentrations, with complete loss of cell structure and decrease of cellular size at 5 and 10 µM. 

## 3. Discussion

The use of nanotechnology in this type of concept aims to overcome current free drug limitations by increasing drug bioavailability and circulation stability, and decreasing peripheral metabolization and toxicity. Taking this into consideration, different optimized NLC formulations were prepared loaded with the respective CNS drugs (TAC, TCP, or RAS), Lys(N_3_)-MAP, CNS-MAP conjugates (TAC-MAP, TCP-MAP, RAS-MAP), and dual encapsulation of Lys(N_3_)-MAP and CNS drugs. The main NLC composition used Compritol 888 ATO as the solid lipid component, 25% of Transcutol HP as the liquid lipid component, and 3% of Tween 80 as the surfactant. All NLC formulations prepared demonstrated a particle size distribution below 160 nm and a polydispersity index below 0.3 (Table 1). Particle size analysis showed that incorporation of drug-MAP conjugates and co-encapsulation of CNS drugs and Lys(N_3_)-MAP did not contribute to particle size increase on NLC formulations when compared to CT25 blank (Table 1). A similar outcome was observed on all NLC zeta potential. Preliminary solubility studies conducted prior to the NLC formulation demonstrated that TAC, TCP, and RAS had a maximum solubility level in liquid lipid Transcutol HP when compared with the other lipids tested. This will contribute to ensure an enhanced encapsulation efficiency (EE) of the drug inside the NLC, which is crucial for enhancing drug efficacy. The overall EE and DL demonstrated higher levels on RAS related NLC formulations than for the others CNS drugs used, most likely to be related to lower water solubility associated with RAS. Even though extensive NLC analysis was evaluated, it is important that in future studies with an in vivo model that a more in-depth analysis be conducted on NLC, such as particle morphology and particle–particle interactions using other available techniques [29,30]. 

To assess and validate the enhanced drug-MAP conjugates efficacy on tumor cell toxicity with the use of nanoparticles, we conducted two cytotoxicity studies (MTT and SRB assays) and microscope cellular visualization. In the first part of the paper, we confirmed the toxic activity against two cell lines (Caco-2 and SH-SY5Y) of chosen peptides and the new synthesized drug-peptide conjugates. Then, we evaluated the differences in their cytotoxic activity when encapsulated in NLC, co-administrated and co-encapsulated in the NLC. The results of free peptide administration demonstrated, as expected, a high cytotoxic activity and strong membrane lytic proprieties of peptide MAP against both cell lines at 10 µM, a result similar to the value already described in the literature [31]. The treatment with lysine-modified MAP (Lys(N_3_)-MAP) demonstrated higher toxicity than MAP against the Caco-2 cell line at 5 µM (Figure 1). In addition, all synthesized drug-MAP conjugates demonstrated that membrane lytic proprieties of MAP were maintained even when conjugated to CNS drug and its respective toxic activity was observed at the same concentrations (Figure 1). Furthermore, the data collected from cytotoxicity assays demonstrated that CT25 blank, CT25 TAC, CT25 TCP, and CT25 RAS were relatively safe to both cell lines used. Even though some toxicity was observed with a decrease of around 20% of cell viability, this value was expected from our previous studies. This experimental result is related to the use of Tween 80 as a surfactant in the NLC formulation [18,32,33]. After encapsulation of drug–MAP conjugates and Lys(N_3_)-MAP, the cytotoxic assays and microscope evaluation demonstrated a significant decrease in cell viability in both cell lines and severe cell morphological changes after treatment when compared with free drug-MAP and free Lys(N_3_)-MAP at the lowest concentration. To ensure that nanoparticles were more efficient to deliver the toxic compound and thus contributing to enhancement of cytotoxic activity, we conducted a co-administration assay with the treatment of both CT25 blank and free drug–MAP on the tumoral cells. The results from co-administration only demonstrated toxicity at higher concentrations. This study showed that encapsulation of drug-conjugates did not compromise their toxic effect and showed that was crucial to ensure higher drug efficacy at lower concentrations. In addition, we also prepared a co-encapsulated NLC to evaluate the toxic capability of CNS drug and Lys(N_3_)-MAP combined but not covalently bound. The cytotoxic assays and microscope analysis showed less toxic activity when compared with NLC encapsulated with drug–MAP conjugate.

In conclusion, we were able to successfully synthesize a TCP–MAP conjugate and demonstrate its antiproliferative activity against two distinct cancer cell lines, Caco-2 and SH-SY5Y. Moreover, with this work we were able to encapsulate CNS drugs such as RAS, TAC, and TCP; drug-MAP conjugates; and amphipathic peptide Lys(N_3_)-MAP and co-encapsulate both CNS drug and Lys(N_3_)-MAP into the NLC matrix. The overall cytotoxic assays confirmed the potential use of nanoparticles as carrier systems to successfully deliver therapeutic compounds not only useful against cancer but which also have potential in neurodegenerative diseases. In the future it will be important to evaluate the mechanisms behind drug–MAP conjugates anticancer activity with cells. 

## 4. Materials and Methods

### 4.1. Materials

Tacrine hydrochloride (TAC; 9-amino-1,2,3,4-tetrahydroacridine hydrochloride hydrate), Tranylcypromine (TCP; (±)-trans-2-Phenylcyclopropylamine) and Rasagiline mesylate (RAS; N-Propargyl-1(R)-aminoindan methanesulfonate) were acquired from Sigma Aldrich (St Louis, MO, USA). Diethylene glycol monoethyl ether (Transcutol HP^®^) and glyceryl dibehenate (Compritol^®^ 888 ATO) were a kind gift from Gattefossé (France). Polysorbate 80 (Tween 80^®^) was purchased from J. Vaz Pereira S.A. (Portugal). From Sigma-Aldrich (St Louis, MO, USA) were purchased 3-(4.5-dimethyl-2-thiazolyl)- 2,5-dipheny-2H-tetrazolium bromide (MTT), sulfarhodamine B sodium (SRB), and phosphate buffer solution (PBS - pH 7.4). Caprylic/capric triglycerides (Miglyol^®^ 812) were a gift from Sasol Olefins & Surfactants GmbH (Hamburg, Germany). Dimethyl sulfoxide (DMSO) was acquired from Millipore-Sigma. Fluorescamine was purchased from Sigma-Aldrich. For the viability studies, neuroblastoma cell line (SH-SY5Y, ATCC^®^ CRL-2266^TM^) and colorectal adenocarcinoma cell line (Caco-2, ATCC^®^ HTB-37™) were used. Cell culture medium and penicillin-streptomycin solution were acquired from Sigma-Aldrich and the rest of the cell culture supplies were acquired from Millipore-Sigma.

### 4.2. TCP-MAP Synthesis

**Figure 17:**
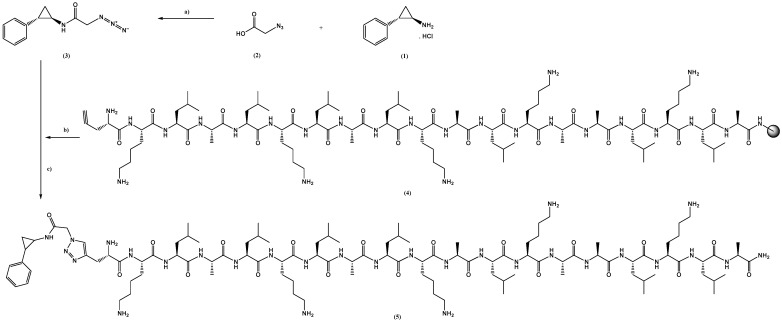
Representation of the methodology [a)–d)] applied for the synthesis of the TCP–MAP conjugate (5), from (1) TCP, (2) linker, (3) 2-PCPA-linker and (4) Pra-MAP. a) Reaction to link TCP to the linker. b) Pra-MAP expose to conjugate with TCP with linker through click reaction; c) final desprotection and isolation of TCP-MAP conjugate.

MAP (KLALKLALKALKAALKLA-NH_2_), TAC-MAP, and RAS-MAP were previously synthetized by our research group as described in refs. [17,28]. Briefly, MAP was synthetized through Fmoc/tBu solid-phase peptide synthesis (SPPS) methodologies assisted with microwave (MW) energy, using a Liberty Microwave Peptide Synthesizer. Conjugation with TAC or RAS was achieved through “click chemistry”—a classical copper-catalyzed azide–alkyne cycloaddition (CuAAC) reaction. In this article, we proceed to the new conjugation of TCP into MAP peptide sequence using the same chemical reaction abovementioned, CuAAC (Figure 17). Details for preparation of TCP-MAP are in the Supporting Materials (Synthesis of TCP-linker-N_3_, Synthesis of MAP and Synthesis of TCP-MAP, Figures S1–S9). 

### 4.3. TCP Solubility Studies

The solubility of TCP was determined in Compritol 888 ATO, Transcutol HP, and Miglyol to ensure encapsulation of TCP into the core of the NLC. Briefly, the solid lipid Compritol 888 ATO was melted at a temperature 10 °C above its melting point (80 °C), in a controlled-temperature water bath, while the liquid lipids Miglyol and Transcutol HP were ready to use at room temperature. Increasing amounts of TCP were successively added, with stirring, until saturation of the lipid was achieved. The saturation point occurred when the excess of solid TCP persisted for more than 8 h. Each determination was carried out in triplicate (n = 3).

### 4.4. Preparation of NLC 

TCP-loaded, TCP + MAP-loaded, and TCP-MAP-loaded NLC were prepared using a modification of a hot high-shear homogenization (HSH) method previously described in refs. [18,32]. Briefly, 300 mg of Compritol 888 ATO was melted at a temperature 10 °C above its melting point (80 °C). TCP, TAC, RAS, Drug MAP conjugates and L-(N_3_)-MAP were dissolved in the liquid lipid Transcutol HP (at theoretical concentrations of 25% *w*/*w*), which was added to the molten Compritol 888 ATO. A hot aqueous phase consisting of 10 mL ultra-purified water with 3% of Tween 80 was added to the lipid phase under high-shear homogenization at 12,300 rpm for 10 min on a Silverson SL2 (UK), in a water bath to maintain the temperature. The NLC dispersion was then cooled in an ice bath with gentle stirring for 5 min. Each formulation was carried out in triplicates (n = 3). The final dispersion was sealed and stored at 4 °C until further use.

### 4.5. Characterization of NLC 

#### 4.5.1. Particle Size and Surface Change

Particle size distribution was analyzed by photon correlation spectroscopy using Zetasizer Nano S (Malvern Instruments, Malvern, UK). Samples were placed in polystyrene cuvettes, and their size was measured at a 173° scattering angle at 25.0 ± 0.1 °C. All the results were expressed as average particle size and polydispersity index (PI). Through nanoparticle electrophoretic mobility (previously diluted with filtered, purified water), zeta potential was calculated using a Zetasizer Nano Z (Malvern Instruments, UK). For all measurements, at least three replicates’ samples were determined.

#### 4.5.2. Encapsulation Efficiency

The entrapment efficiency (EE) and drug loading (DL) for all the NLC formulations prepared were determined using one indirect and two direct methods. First, the free drug, i.e., unassociated with the NLC, was separated from the particles using centrifugation (Amicon ultra centrifugal filter units; ultra-15, MWCO 100 Kda, Sigma-Aldrich, Algés, Portugal). Briefly, 1 mL of the sample was kept in the upper chamber compartment of the ultra-centrifuge tube and centrifugated at 12,000 rpm for 15 min. The collected sample in the lower chamber was quantified using high-pressure liquid chromatography (HPLC). The second, unencapsulated with drug and peptide was separated from the NLC by size exclusion chromatography using Sephadex G-25/ PD-10 columns. Encapsulated drug and peptide to NLC was calculated after dissolving the NLC with acetonitrile (1:1) followed by a centrifugation (30 min at 12,300 rpm) step (Hettich Universal 320R, Föhrenstr, Germany), promoting the lipid-phase precipitation. After centrifugation, the encapsulated compounds remained in the supernatant, and the drugs were measured by HPLC and the peptide conjugates measured by a fluorometric assay. Briefly, 50 µL of fluorescamine (3 mg/mL) was added to 150 µL solution with 50 µL of aliquot supernatant and 100 µL of PBS at pH 10 in a black 96-well plate. The fluorescent intensity was measured using a fluorescent plate reader equipped with a 390 nm excitation filter and a 475 nm absorption filter. The concentration of the samples was calculated through each peptide calibration curve. In addition, entrapment efficiency and drug loading were determined using the following equations:

$$Encapsulation\;efficiency\;\left(\%\frac{w}{w}\right)=\frac{\left(w1-w2\right)}{w1}\times 100$$

$$Drug\;loading\;\left(\%\frac{w}{w}\right)=\frac{\left(w1-w2\right)}{w3}\times 100$$

where *w*1 is the amount of drug added in the NLCs, *w*2 is the amount of free drug, and *w*3 is the amount of lipid.

### 4.6. In Vitro Cell Viability Studies 

The cytotoxicity of all NLC formulations against SH-SY5Y and Caco-2 cell lines were assessed by MTT, SRB, and microscope visualization. 

#### 4.6.1. MTT Assay

The MTT assay was conducted to determine metabolic activity of cells and assess cellular viability. Cells were maintained in culture incubated with DMEM further supplemented with 10% of heat-inactivated fetal bovine serum (FBS) and 1% penicillin and streptomycin at 37 °C in a controlled atmosphere of 5% CO_2_ (according to the ATCC recommendations). Before the experiment, SH-SY5Y cells and Caco-2 cell line were seeded in sterile, flat-bottom 96-well plates at the density of 18,000 cells per well and 6000 cells per well, respectively, and incubated for 24 h. In the next day, the medium was replaced with increasing concentrations of free TAC, free RAS, free TCP, free TAC-MAP, free RAS-MAP, free TCP-MAP, and drug and peptide–drug conjugates-loaded NLCs. Then the cells were incubated for 24 h and both negative and positive control were included with incubation with culture media and sterilized water (5:1) and DMSO 10% to promote the cell lyses, respectively. After incubation, all media was aspirated and replaced with 100 μL of MTT solution (at 0.5 mg/mL). Then, cells were incubated for 3 h in the dark. After this, the medium was aspirated and 100 μL DMSO was added to dissolve the formazan crystals. The absorbance was measured at 570 nm in a microplate reader (Synergy HT, Biotech Instruments inc., Winoosk, USA and Tecan Infinite M200, Tecan group Ltd., Männedorf, Switzerland). All experiments were performed in triplicates and the relative cell viability (%) was compared to control cells and calculated using the following equation:

$$Cell\;viability\;\left(\%\;of\;sample\right)=\frac{Abs\;sample}{Abs\;control}\times 100$$

#### 4.6.2. SRB Assay 

Cell viability assay of all formulations was also determined by SRB assay. For this, the same conditions and methods described above for MTT assay were used. After cells were treated with all NLC formulations and respective controls, the cells were fixed with 100 μL of ice-cold 10% trichloroacetic acid (TCA) per well for 1 h at 4 °C. Then TCA was aspirated and allowed to dry before adding 100 μL of 0.4% SRB solution. After 1 h, the plates were washed with running tap water and air-dried. The incorporated dye was solubilized by the addition of 200 μL of 10 mM tris buffer per well. After that, the absorbance was measured at 510 nm in a microplate reader (Synergy HT, Biotech Instruments Inc., Winoosk, VT, USA and Tecan Infinite M200, Tecan group Ltd., Männedorf, Switzerland). All experiments were performed in triplicates.

#### 4.6.3. Cell Morphology Visualization

After the treatments, cell morphology and growth were assessed by contrast phase microscope Lionheart FX (Biotech, USA) with use of Gen5 software (Biotech, USA) and a Leica DMI 6000B microscope equipped with a Leica DFC350 FX camera and then analyzed with the Leica LAS X imaging software (v3.7.4). 

### 4.7. Statistical Analysis 

Statistical analysis of the experimental data was performed using a one-way analysis of variance (one-way ANOVA) and differences between groups were tested by a two-way ANOVA with GraphPad Prism version 8.0 (GraphPad Software, San Diego, CA, USA). Data were expressed as means ± SD or 95% confidence interval. A *p* < 0.05 value was considered significant.

## Figures and Tables

**Table 1:** Physicochemical properties of all NLCs formulated freshly (mean ± SD, n = 2 or 4).

NLC	NLC Composition	PS (nm)	PI	ZP (mV)	EE %	DL %
CT25 B	Lipids: 75% C and 25% TE Surfactant: 3% T80	122.0 ± 31.5	0.305 ± 0.07	−4.1 ± 3.6	--	--
CT25 Lys(N_3_)-MAP	Lipids: 75% C and 25% TE Surfactant: 3% T80	112.1 ± 9.5	0.277 ± 0.09	−2.0 ± 0.1	12.6 ± 59.3	2.0 ± 7.1
CT25TAC	Lipids: 75% C and 25% TE Surfactant: 3% T80	117.2 ± 55.0	0.290 ± 0.06	−4.4 ± 5.3	35.5 ± 22.6	1.6 ± 0.3
CT25TAC-MAP	Lipids: 75% C and 25% TE Surfactant: 3% T80	98.0 ± 11.6	0.251 ± 0.03	−4.3 ± 3.9	18.2 ± 2.3	3.7 ± 0.9
CT25 Lys(N_3_)-MAP + TAC	Lipids: 75% C and 25% TE Surfactant: 3% T80	122.0 ± 1.1	0.236 ± 0.02	−1.0 ± 0.3	2.7 ± 50.9	2.0 ± 10.1
CT25 TCP	Lipids: 75% C and 25% TE Surfactant: 3% T80	164.1 ± 43.0	0.362 ± 0.05	−3.5 ± 5.6	26.0 ± 8.0	1.6 ± 0.7
CT25 TCP-MAP	Lipids: 75% C and 25% TE Surfactant: 3% T80	92.1 ± 3.2	0.272 ± 0.03	−1.7 ± 0.4	14.2 ± 1.4	1.7 ± 0.2
CT25 Lys(N_3_)-MAP + TCP	Lipids: 75% C and 25% TE Surfactant: 3% T80	128.3 ± 6.5	0.247 ± 0	−0.4 ± 0.1	18.7 ± 0.0	1.9 ± 0.7
CT25 RAS	Lipids: 75% C and 25% TE Surfactant: 3% T80	114.8 ± 0.0	0.274 ± 0.04	−3.6 ± 3.6	63.1 ± 26.0	0.3 ± 0.3
CT25 RAS-MAP	Lipids: 75% C and 25% TE Surfactant: 3% T80	129.0 ± 3.1	0.281 ± 0.03	−1.1 ± 0.1	58.3 ± 39.2	5.2 ± 1.4
CT25 Lys(N_3_)-MAP + RAS	Lipids: 75% C and 25% TE Surfactant: 3% T80	88.0 ± 5.3	0.233 ± 0	−2.0 ± 0.3	35.4 ± 29.8	3.8 ± 2.8

PS: Mean particle size; PI: polydispersity index; ZP: Zeta potential; EE: encapsulation efficiency; DL: drug loading; C: Compritol ATO 888; TE: Transcutol HP; T80: Tween 80; B: Blank.

## Supplementary Materials

The following are available online at https://www.mdpi.com/article/10.3390/ijms23137109/s1, Synthesis of TCP-linker-N3, Synthesis of MAP and Synthesis of TCP-MAP.
