# Supplementary material for: New Peptide Functionalized Nanostructured Lipid Carriers with CNS Drugs and Evaluation Anti-proliferative Activity
Source: Int J Mol Sci. 2022 Jun 26;23(13):7109. doi: 10.3390/ijms23137109 (PMC9266917; doi:10.3390/ijms23137109)
Supplement: Supplementary file 1 [file ijms-23-07109-s001.zip › ijms-1744386-supplementary.pdf]

International Journal of Molecular Sciences

MDPI

Supplementary Materials: New peptide functionalized nanostructured lipid carriers with CNS drugs and evaluation anti-proliferative activity

Sara Silva [1,2,3], Joana Marto [3], Lídia M. Gonçalves [3], Diana Duarte [1,2], Salomé Soares [4,5], Francisco Vasques-Nóvoa [6,7], António J. Almeida [3] and Nuno Vale [1,8,9,*]

1 OncoPharma Research Group, Center for Health Technology and Services Research (CINTESIS), Rua Doutor Plácido da Costa, 4200-450 Porto, Portugal;
2 Faculty of Pharmacy, University of Porto, Rua de Jorge Viterbo Ferreira 228, 4050-313 Porto, Portugal;
3 Research Institute for Medicines (iMed.ULisboa), Faculty of Pharmacy, University of Lisbon, Avenida Professor Gama Pinto, 1649-003, University of Lisbon, Portugal;
4 LSRE-LCM - Laboratory of Separation and Reaction Engineering – Laboratory of Catalysis and Materials, Faculty of Engineering, University of Porto, Rua Doutor Roberto Frias, 4200-465 Porto, Portugal;
5 ALiCE - Associate Laboratory in Chemical Engineering, Faculty of Engineering, University of Porto, Rua Doutor Roberto Frias, 4200-465 Porto, Portugal;
6 Cardiovascular R&D Center, Faculty of Medicine, University of Porto, Porto, Rua Doutor Plácido da Costa, s/n, 4200-450 Porto, Portugal Portugal;
7 Department of Surgery and Physiology, Faculty of Medicine, University of Porto, Alameda Professor Hernâni Monteiro, Porto, 4200-319, Portugal;
8 Department of Community Medicine, Information and Health Decision Sciences (MEDCIDS), Faculty of Medicine, University of Porto, Rua Doutor Plácido da Costa, s/n, 4200-450 Porto, Portugal
9 CINTESIS@RISE, Faculty of Medicine, University of Porto, Alameda Professor Hernâni Monteiro, 4200-319 Porto, Portugal
* Correspondence: nunovale@med.up.pt; Tel.: +351-220426537

TCP-MAP synthesis

Synthesis of TCP-linker-N_3

TCP or 2-PCPA (2-Tranylcypromine)

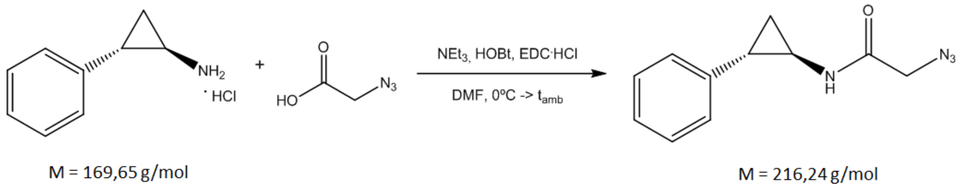

Figure S1 - Schematic representation of conjugate TCP-linker-N_3.

- Azidoacetic acid pre-activation (2 eq.);
- 2 h stirring with 2.5 eq. NEt_3, 2 eq. HOBt and 3 eq. EDC.HCl (ice bath)
- m(2-PCPA)= 100 mg (limitant reagent)
- Solvent: DMF (10 mL) - too much volume
- Reaction time: 20h
- Treatment: liquid-liquid extractions with AcOEt + [NaCl (sat.) + $NaHCO_3$ (sat.) + CH_3COOH 5% + H_2O]
- Purification: Filtration with SiO_2 ('Dry Column Vacuum Chromatography'): eluent Hex/AcOEt 1:3 + AcOEt

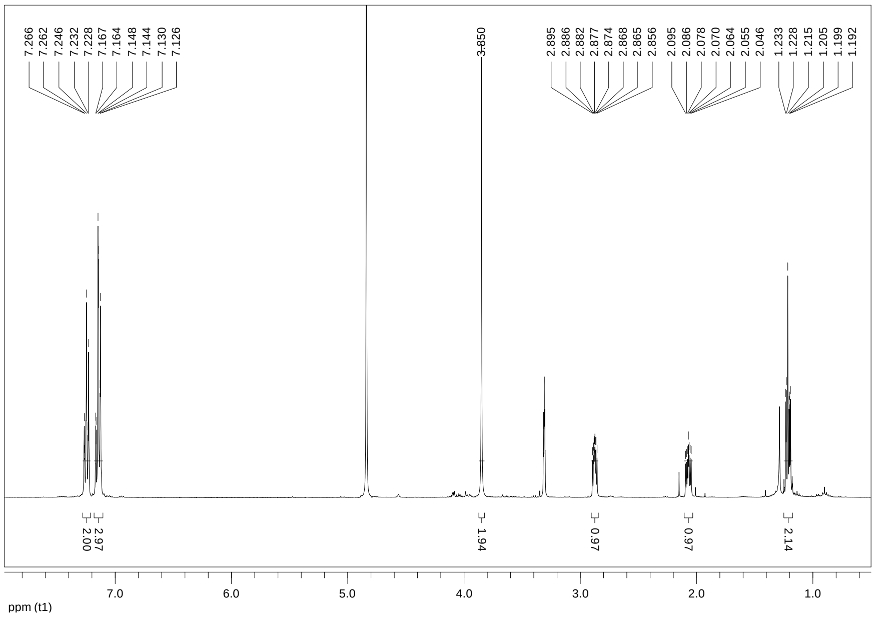

^1H NMR (400 MHz, MeOD) δ(ppm) 7.27 – 7.23 (m, 2H), 7.17 – 7.13 (m, 2H), 3.85 (s, 2H), 2.87 (m, 1H), 2.09 – 2.05 (m, 1H), 1.23 – 1.19 (m, 2H).

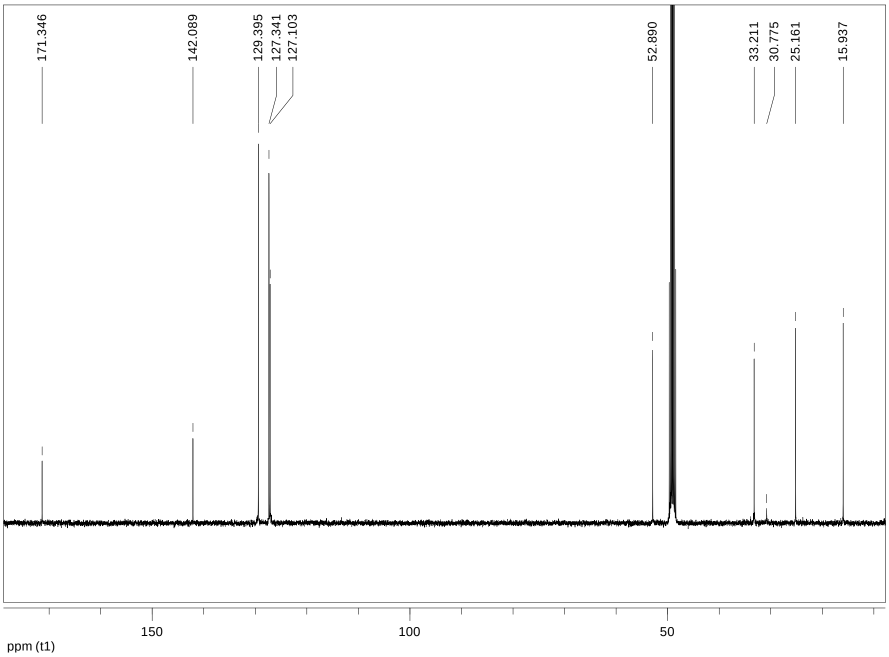

^{13}C NMR (101 MHz, MeOD) δ(ppm) 171.35 (C=O), 142.09 (C), 129.40 (2xCH), 127.34 (3xCH), 127.10 (CH_2), 52.89 (CH_2), 33.21 (CH), 25.16 (CH), 15.94 (CH_2).

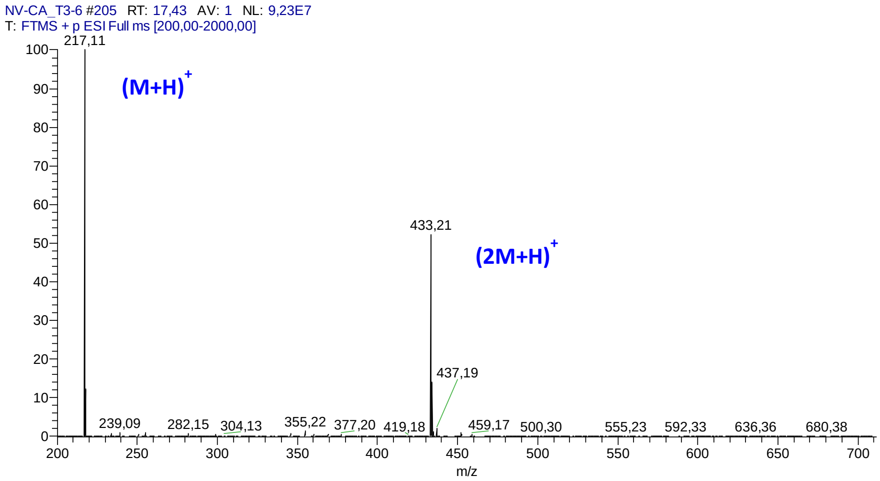

Figure S2 - Mass spectrum (LC-ESI/Orbitrap MS, positive mode) of 2-TCP-linker-N_3.

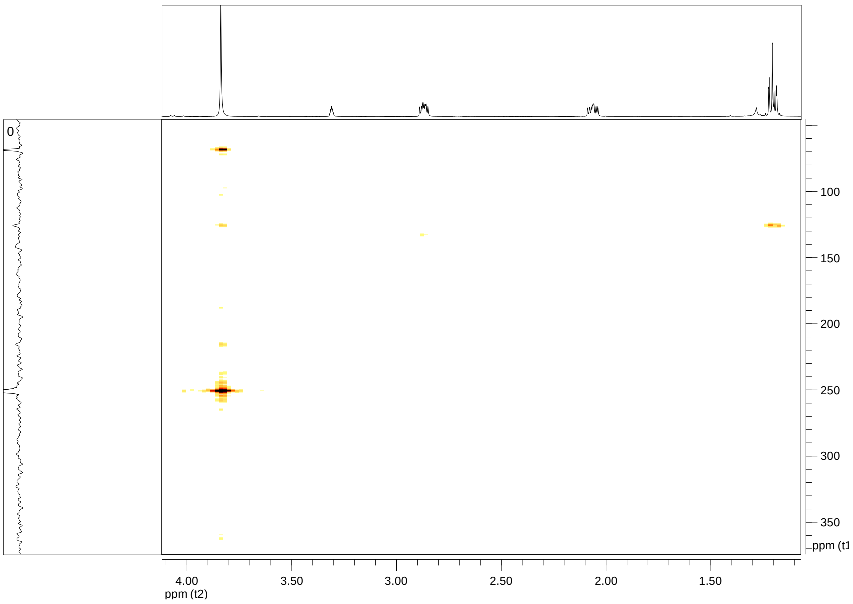

HMBC- ^{15}N (CD_3OD)

- Aspect: yellow oil (when warm), solidifies when cold
- η = 94.4 %

Synthesis of MAP

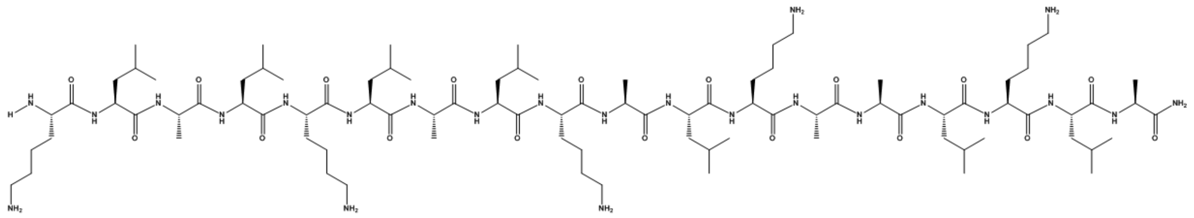

M = 1876.50 g/mol

Figure S3 –Structure of TCP-MAP conjugate.

Structure of peptide MAP.

- Solid support: resin Rink Amide (0.38 mmol/g)
- Synthesis scale: 0.2 mmol
- Protection scheme: orthogonal (Fmoc/tBu)
- Pre-activation: 10 eq. DIEA, 5 eq. amino acid in 5 mL DMF
- Coupling agent: 5 eq. HBTU
- Deprotection: 5 mL 20% piperidine in DMF
- Cleavage: free-thiols cocktail (95 % TFA + 2.5 % H_2O + 2.5 % TIS)

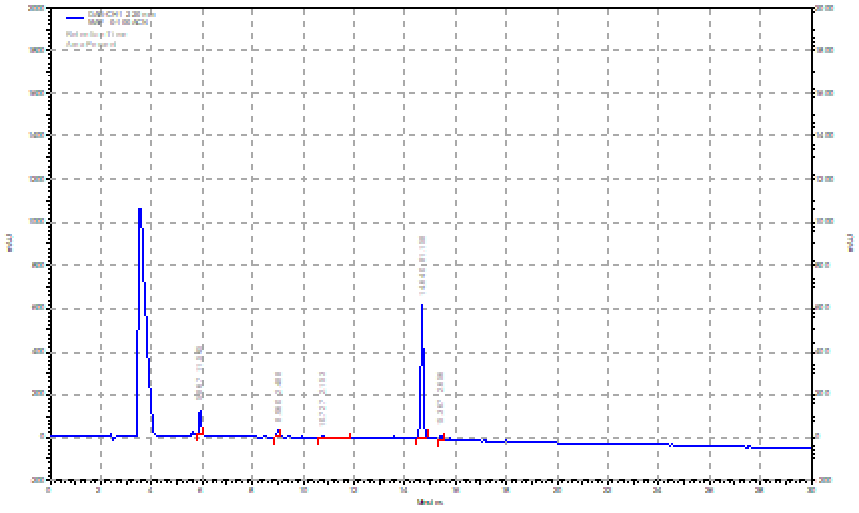

Figure S4 - Chromatogram of the product of the manual synthesis of the MAP, acquired with a HPLC system, with a C18 column, using ACN in water with 0.05% TFA as eluent, in gradient mode (0 – 100%), for 30 minutes, at a flow of 1 mL/min and detection at λ = 220 nm.

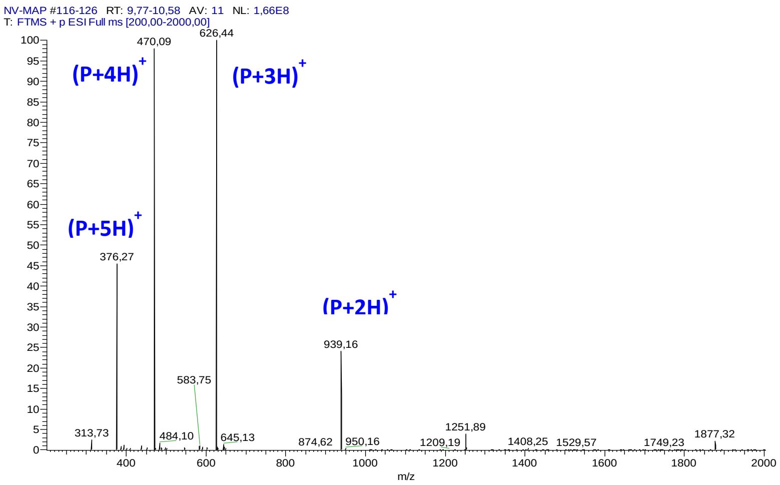

Figure S5 - Mass spectrum (LC-ESI/Orbitrap MS, positive mode) of the peptide MAP.

- Purification: 20-50 % ACN
- Purity: 100 %

Synthesis of Pra-MAP:

- Orthogonal (Fmoc/tBu)
- Pre-activation: 10 eq. DIEA, 5 eq. amino acid in 5 mL DMF
- Coupling agent: 5 eq. HBTU
- Deprotection: 5 mL 20% piperidine in DMF
- Cleavage: free-thiols cocktail (95 % TFA + 2.5 % H_2O + 2.5 % TIS)
- Purity: 100 %

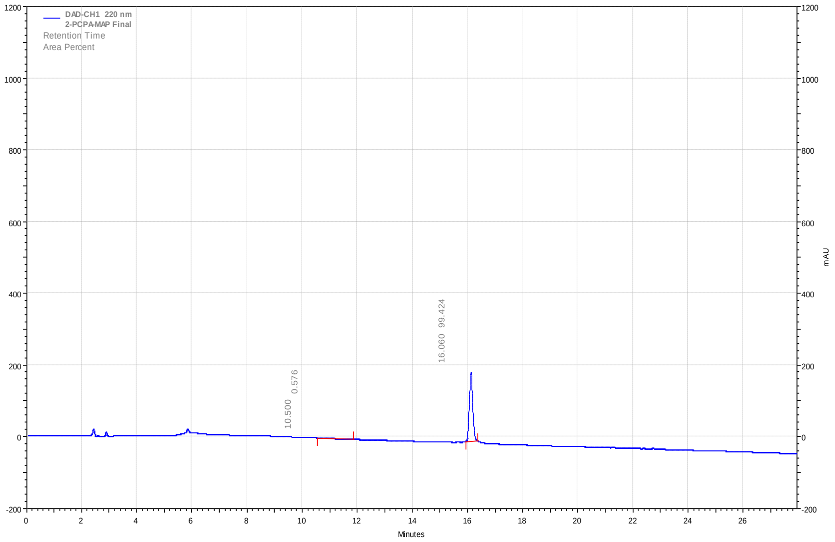

Figure S6 - Chromatogram of the product of the manual synthesis of Pra-MAP, acquired with a HPLC system, with a C18 column, using ACN in water with 0.05% TFA as eluent, in gradient mode (0 – 100%), for 30 minutes, at a flow of 1 mL/min and detection at λ = 220 nm.

Synthesis of TCP-MAP

Click reaction (CuAAC)

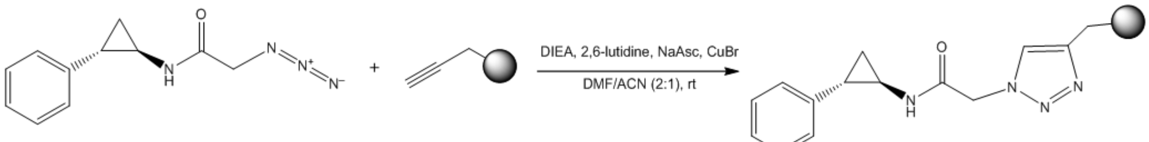

"2-PCPA-linker"
M = 216,24 g/mol

"Pra-MAP-resin"

"2-PCPA-MAP-resin"

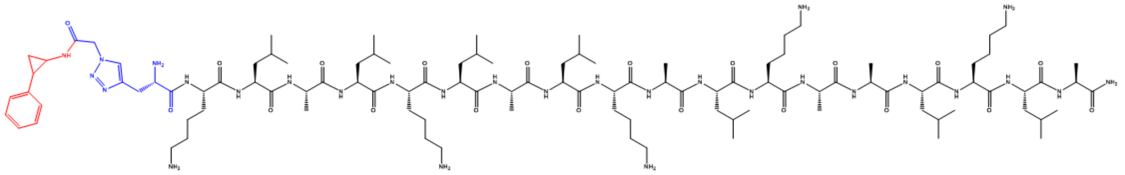

Chemical Formula: $C_{106}H_{187}N_{29}O_{20}$
Exact Mass: 2186,4507
Molecular Weight: 2187,8450

Figure S7 - Schematic representation of conjugate TCP-MAP formation through click reaction; Structure of TCP-MAP conjugate.

- Solid support: resin Rink Amide (0.38 mmol/g)
- Synthesis scale: 0.2 mmol
- 1 eq. "2-PCPA-linker", 10 eq. DIEA, 10 eq. 2,6-lutidine, 1 eq. NaAsc, 1 eq. CuBr
- Solvent: DMF + ACN (2:1, 3 mL)
- Reaction time: 24h
- Treatment: resin wash (filtration) with H_2O, MeOH, DMF and DCM (3x each)
- Cleavage: thiol-free cocktail (95 % TFA + 2.5 % H_2O + 2.5 % TIS)

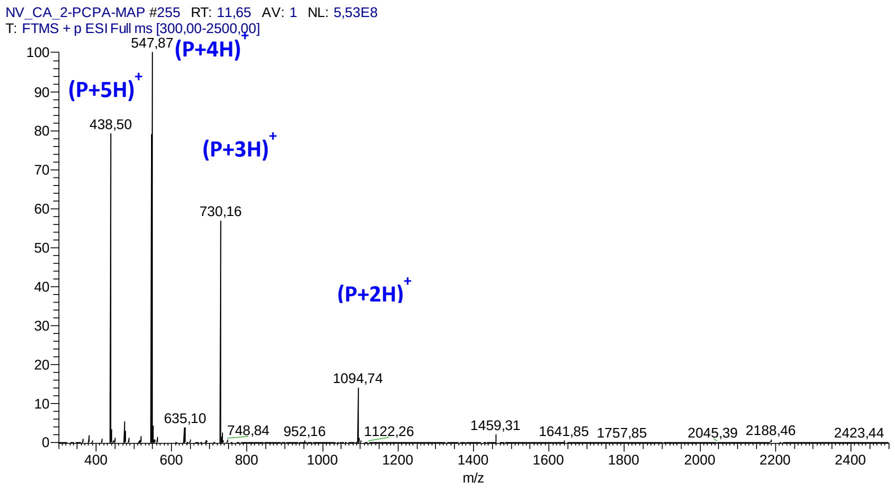

Figure S8 - Mass spectrum (LC-ESI/Orbitrap MS, positive mode) of the peptide TCP-MAP.

- Purification: 20-50 % ACN
- Purity: 100 %

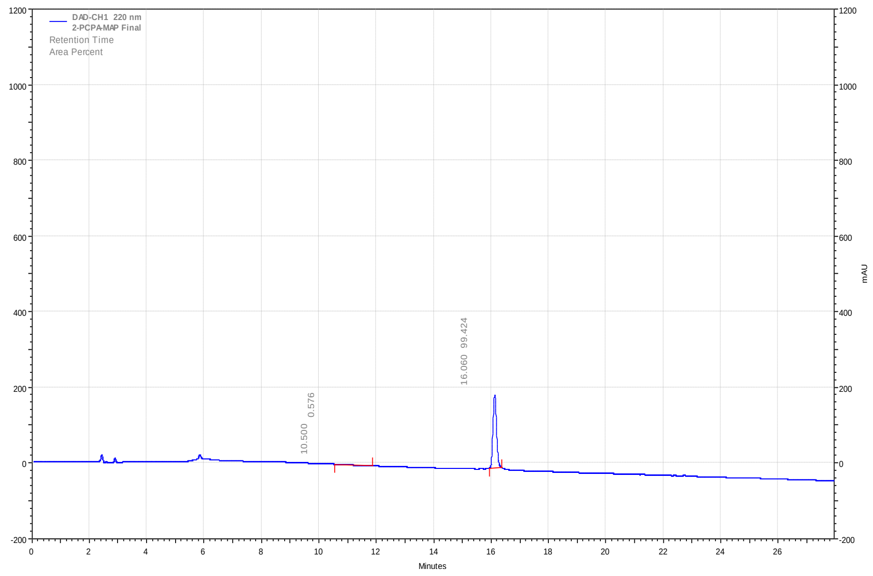

Figure S9 - Chromatogram of the product of the manual synthesis of TCP-MAP, acquired with a HPLC system, with a C18 column, using ACN in water with 0.05% TFA as eluent, in gradient mode (0 – 100%), for 30 minutes, at a flow of 1 mL/min and detection at λ = 220 nm.

In vitro cytotoxicity assays against Caco-2 cell line

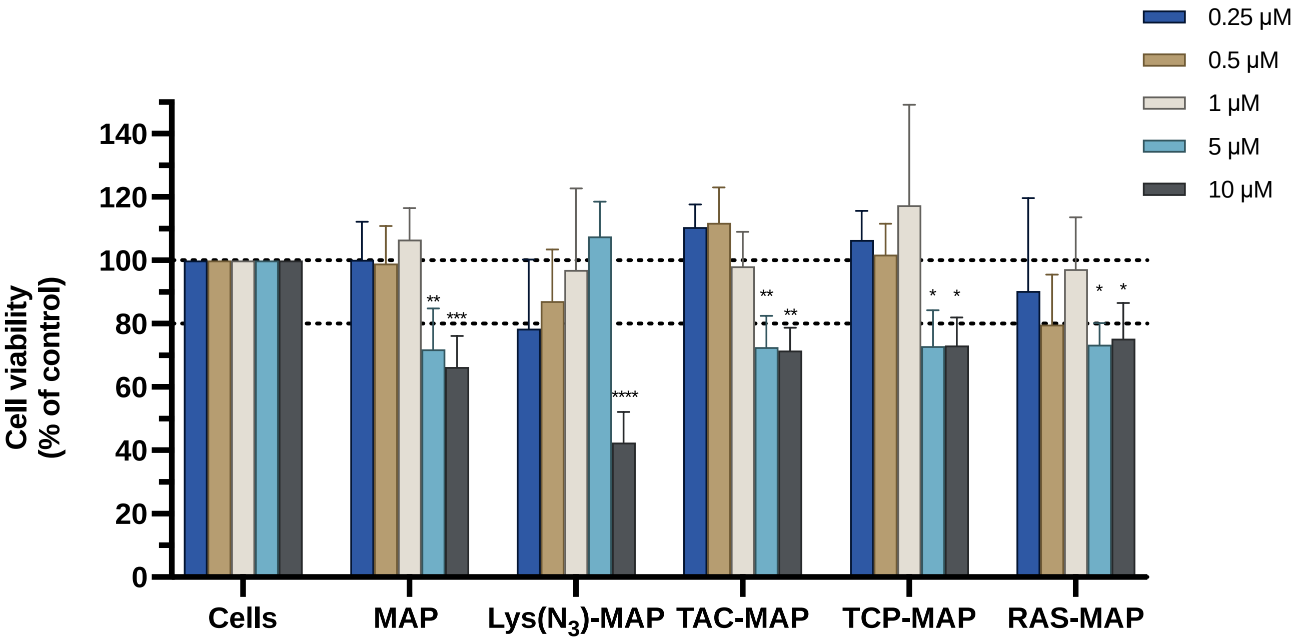

Figure S10 – Relative cell viability of Caco-2 cell line measured by the SRB assay after 24 h incubation. Treatment with free MAP, Lys(N3)-MAP, TAC-MAP, TCP-MAP and RAS-MAP (0.25 μM-10 μM). Results are expressed as mean± SD (n = 3 or 6). Statistical analysis between the control groups was performed using two-way ANOVA with Tukey's multiple comparisons test (* *p* <0.05; ** *p* <0.01; *** *p* <0.001; **** *p* <0.0001).

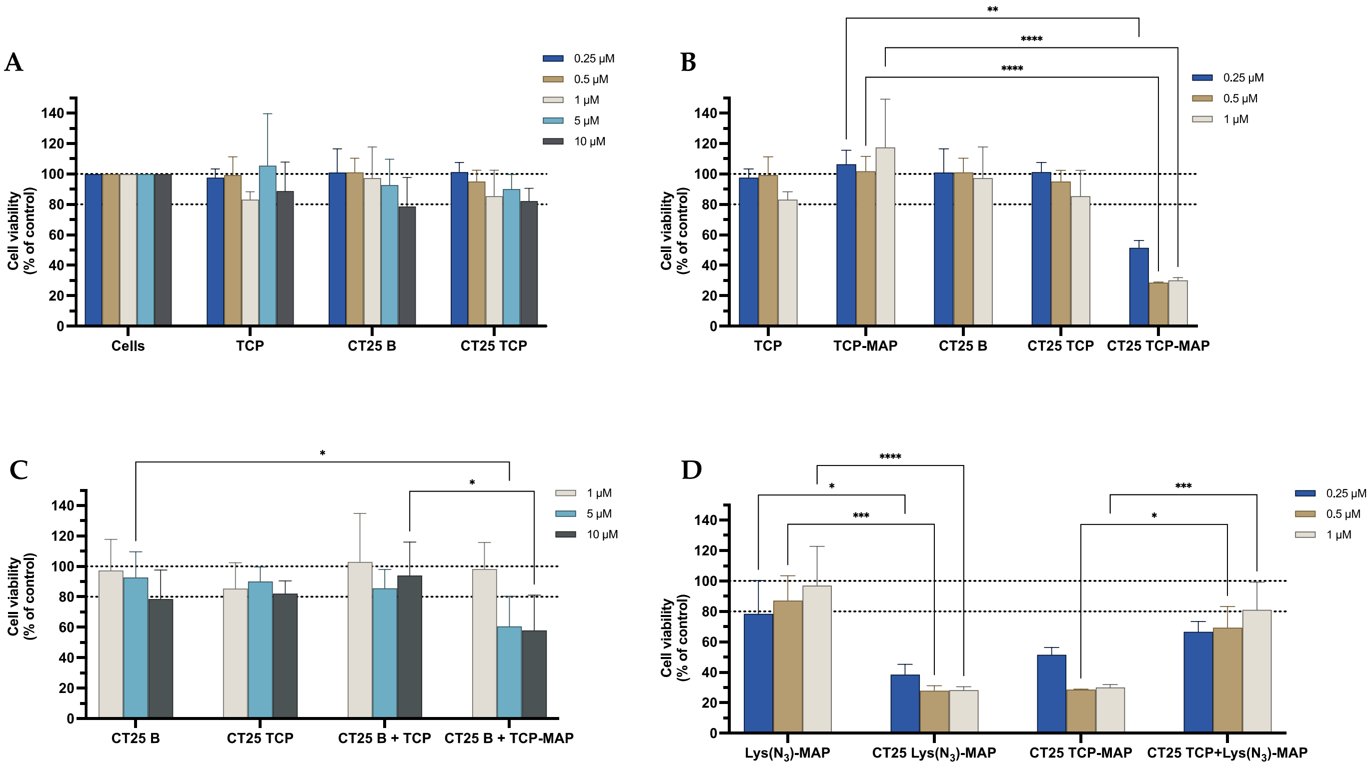

Figure S11 - Relative cell viability of Caco-2 cell line measured by the SRB assay after 24 h incubation. (A) represents NLC safety test with the treatment of free TCP, blank CT25 and TCP loaded CT25 (0.25-10 μM). (B) Represents differences in the cell viability of treatment with free TCP, TCP-MAP, blank CT25, TCP loaded CT25, MAP, TCP-MAP loaded CT25 (0.25-10 μM). (C) Represents co-administration of TCP and TCP-MAP with blank CT25 (1-10 μM). (D) Represents the data obtained from the encapsulation of Lys(N_3)-MAP and co-encapsulation of TCP and Lys(N_3)-MAP in the NLC compared to peptide conjugate. Results are expressed as mean±SD (n = 4). Statistical analysis between the groups was performed using two-way ANOVA with Tukey's multiple comparisons test (* p <0.05; ** p <0.01; *** p <0.001; **** p <0.0001).

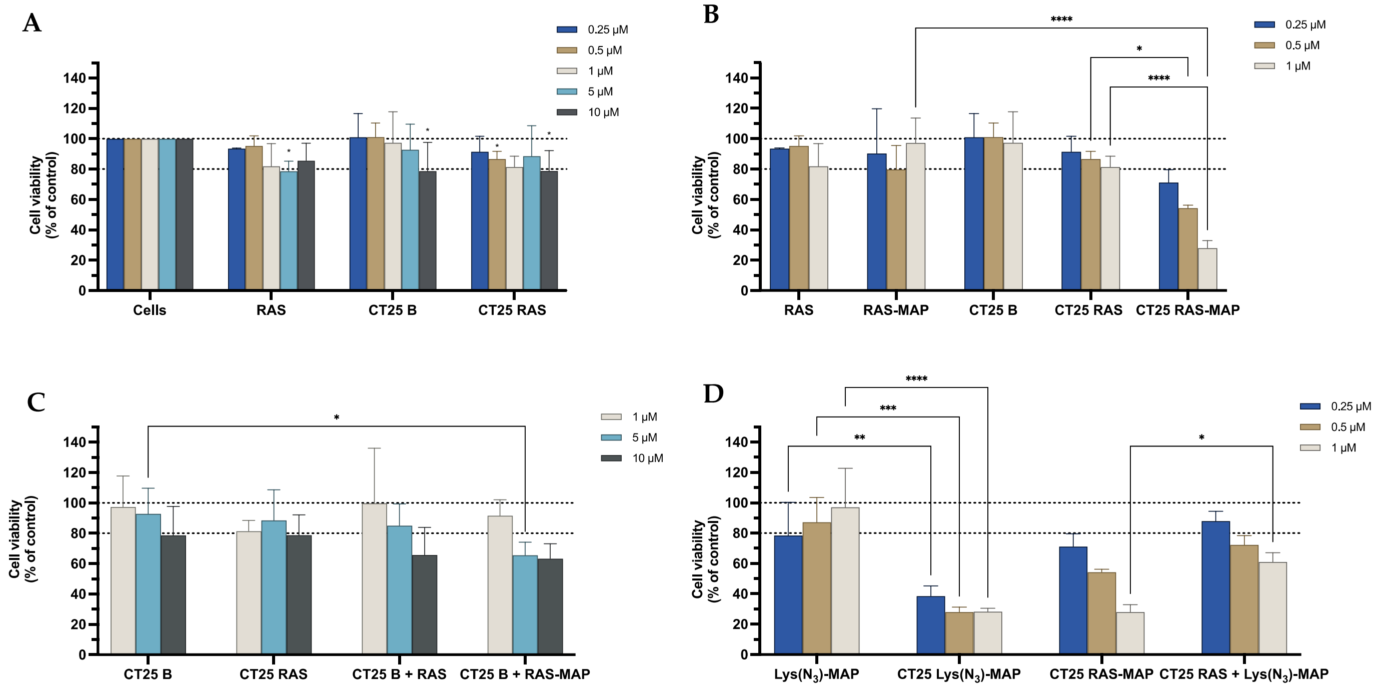

Figure S12 - Relative cell viability of Caco-2 cell line measured by the SRB assay after 24 h incubation. (A) represents NLC safety test with the treatment of free RAS, blank CT25 and RAS loaded CT25 (0.25-10 μM). (B) Represents differences in the cell viability of treatment with free RAS, RAS-MAP, blank CT25, RAS loaded CT25, MAP, RAS-MAP loaded CT25 (0.25-10 μM). (C) Represents co-administration of RAS and RAS-MAP with blank CT25 (1-10 μM). (D) Represents the data obtained from the encapsulation of Lys(N_3)-MAP and co-encapsulation of RAS and Lys(N_3)-MAP in the NLC compared to peptide conjugate. Results are expressed as mean±SD (n = 4). Statistical analysis between the groups was performed using two-way ANOVA with Tukey's multiple comparisons test (* *p* <0.05; ** *p* <0.01; *** *p* <0.001; **** *p* <0.0001).

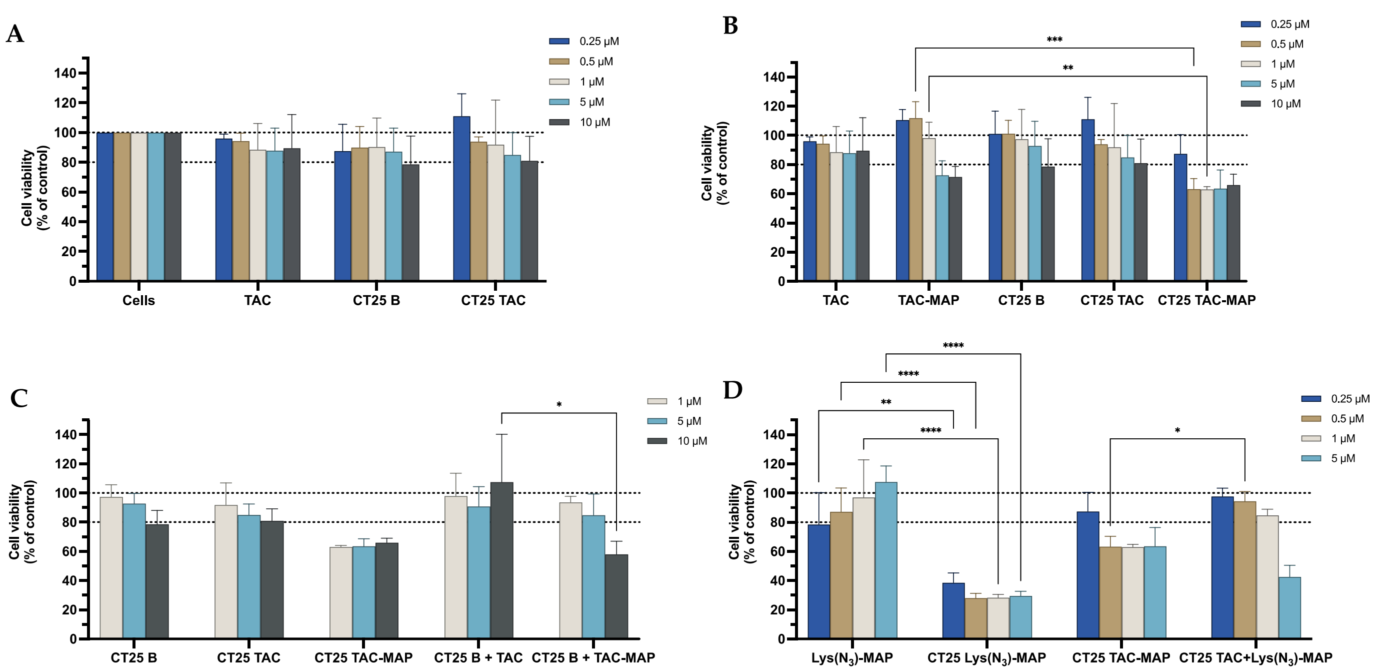

Figure S13 - Relative cell viability of Caco-2 cell line measured by the SRB assay after 24 h incubation. (A) represents NLC safety test with the treatment of free TAC, blank CT25 and TAC loaded CT25 (0.25-10 µM). (B) Represents differences in the cell viability of treatment with free TAC, TAC-MAP, blank CT25, TAC loaded CT25, MAP, TAC-MAP loaded CT25 (0.25-10 µM). (C) Represents co-administration of TAC and TAC-MAP with blank CT25 (1-10 µM). (D) Represents the data obtained from the encapsulation of Lys(N_3)-MAP and co-encapsulation of TAC and Lys(N_3)-MAP in the NLC compared to peptide conjugate. Results are expressed as mean±SD (n = 4). Statistical analysis between the groups was performed using two-way ANOVA with Tukey's multiple comparisons test (* p <0.05; ** p <0.01; *** p <0.001; **** p <0.0001).

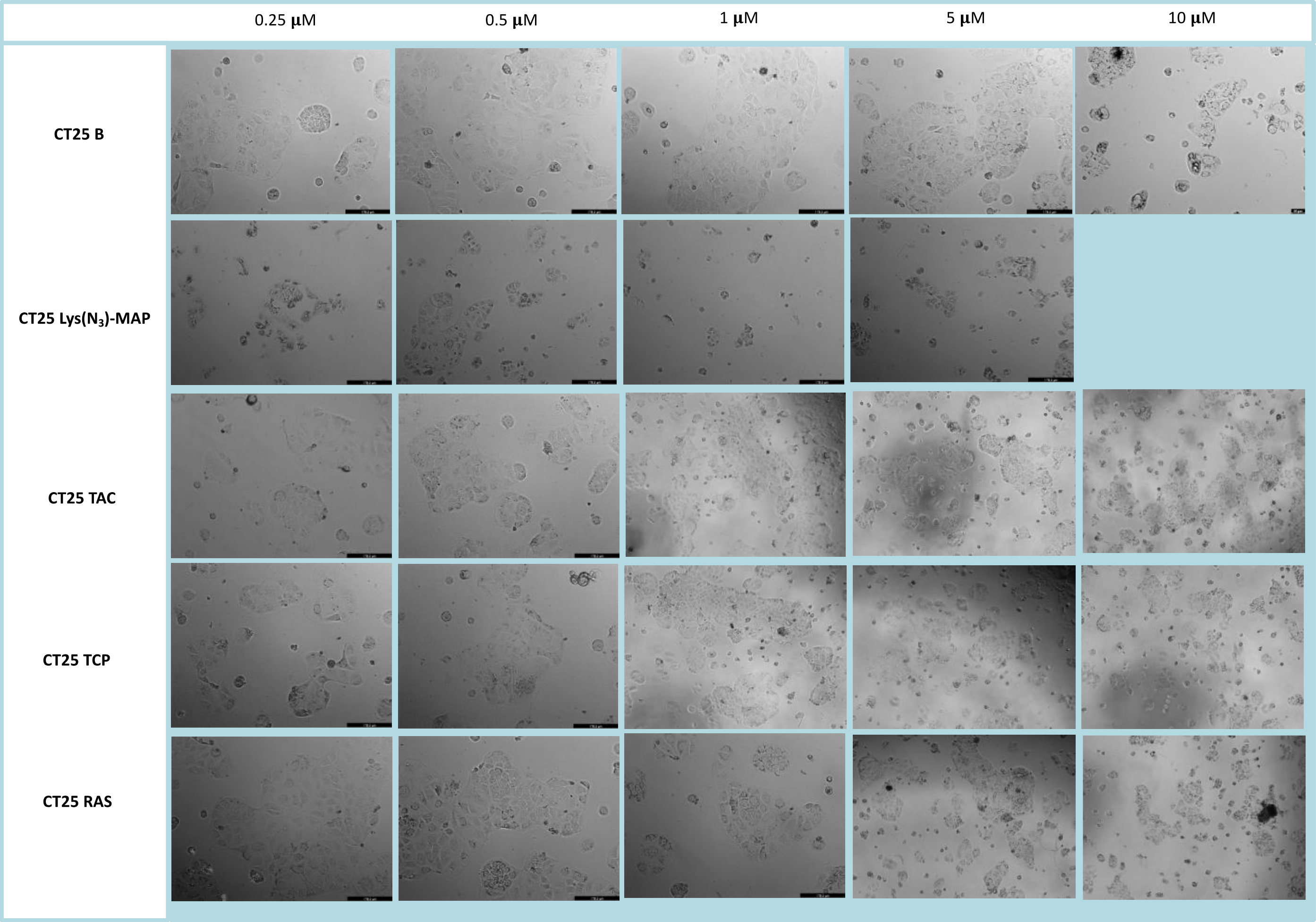

Figure S14 - Microscope cellular visualization through Lionheart FX and Leica DFC350 FX of treatment with free drug and NLC loaded formulations on Caco-2 cell line at a concentration of 0.25, 0.5, 1, 5 and 10 µM.

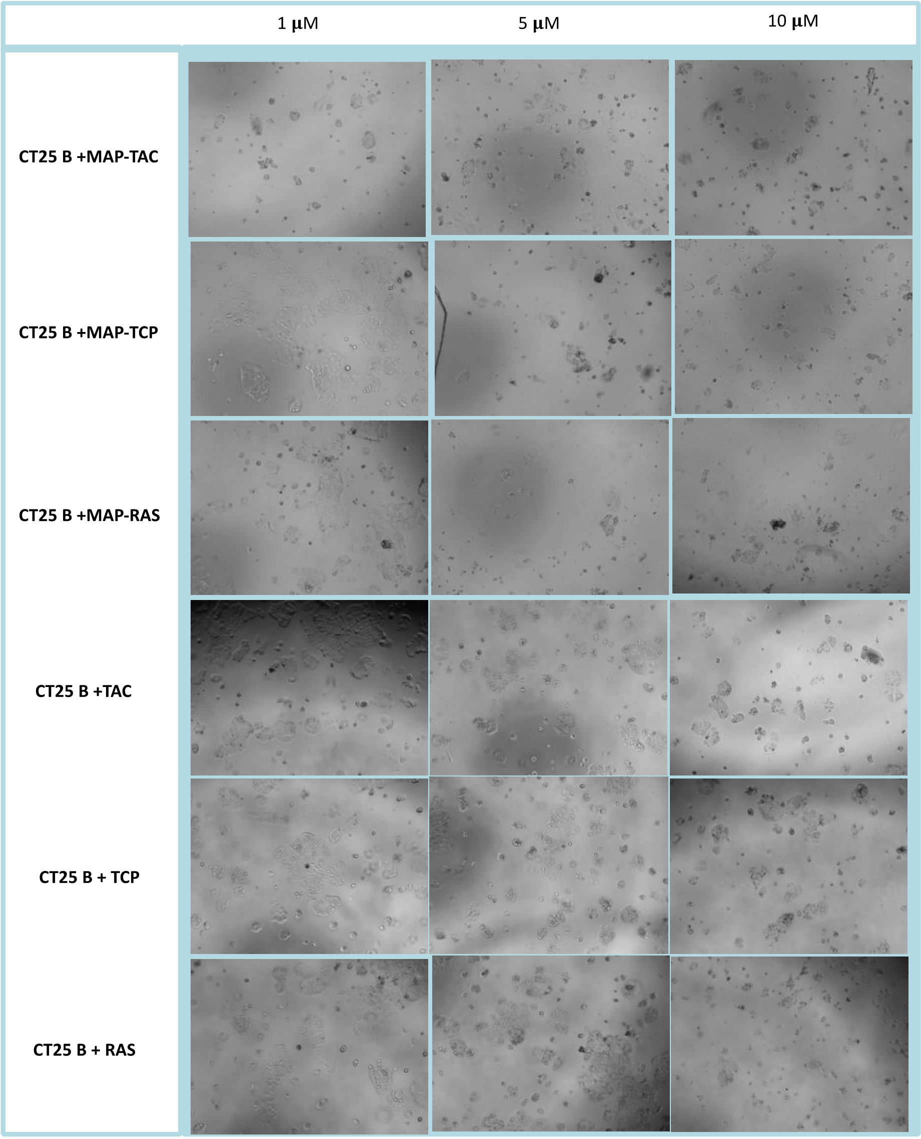

Figure S15 - Microscope cellular visualization through Lionheart FX and Leica DFC350 FX of treatment with free drug and NLC loaded formulations on Caco-2 cell line at a concentration of 1, 5 and 10 µM.

In vitro cytotoxicity assays against SH-SY5Y

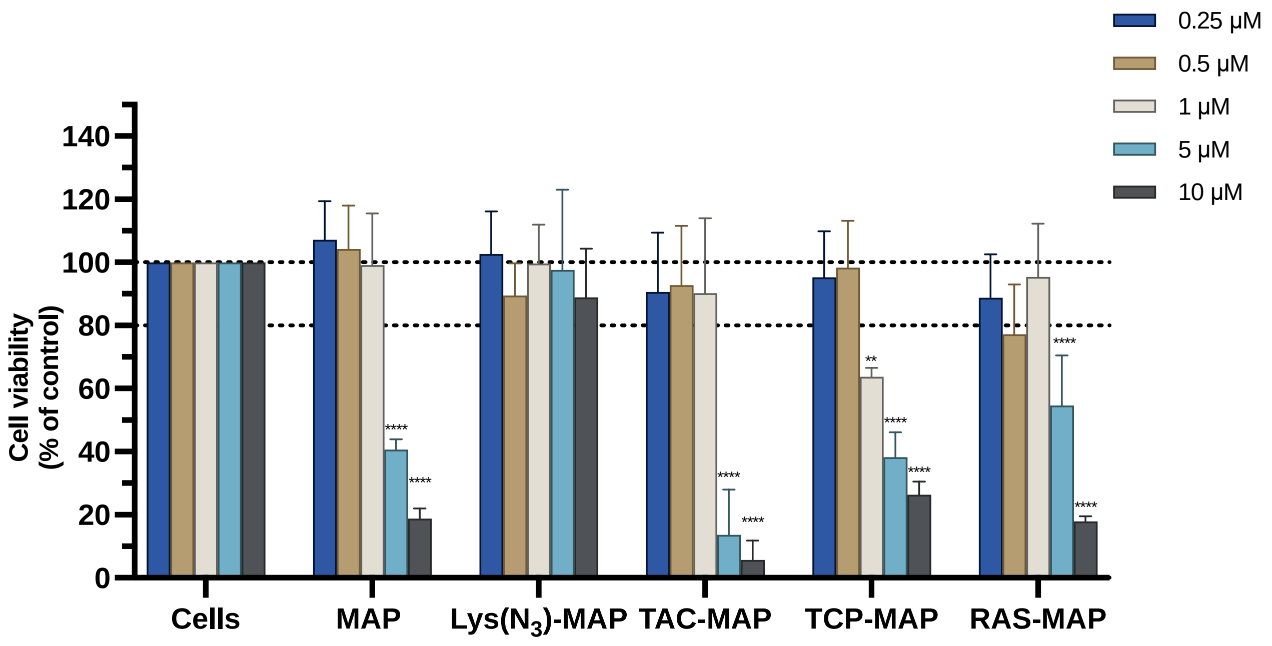

Figure S16 – Relative cell viability of SH-SY5Y cell line measured by the SRB assay after 24 h incubation. Treatment with free MAP, Lys(N_3)-MAP, TAC-MAP, TCP-MAP and RAS-MAP (0.25 μM-10 μM). Results are expressed as mean±SD (n = 3 or 6). Statistical analysis between the control groups was performed using two-way ANOVA with Tukey's multiple comparisons test (** p <0.01; **** p <0.0001).

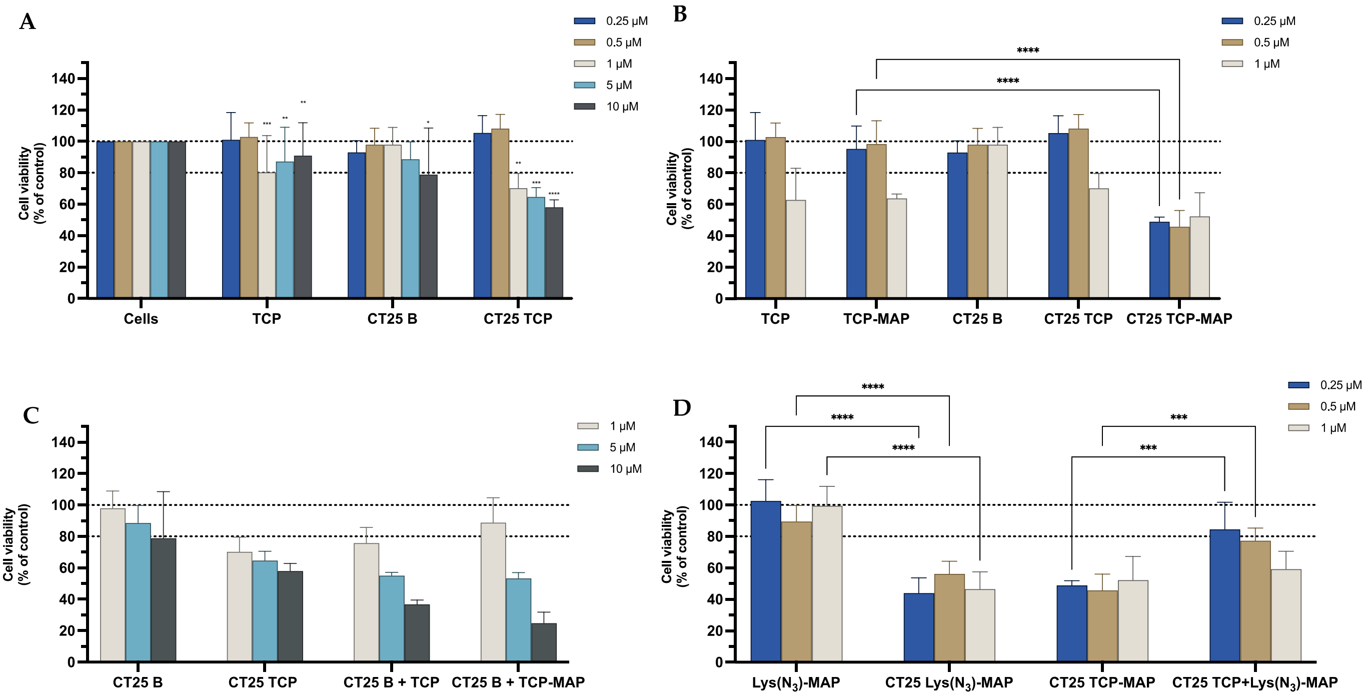

Figure S17 - Relative cell viability of SH-SY5Y cell line measured by the SRB assay after 24 h incubation. (A) represents NLC safety test with the treatment of free TCP, blank CT25 and TCP loaded CT25 (0.25-10 μM). (B) Represents differences in the cell viability of treatment with free TCP, TCP-MAP, blank CT25, TCP loaded CT25, MAP, TCP-MAP loaded CT25 (0.25-10 μM). (C) Represents co-administration of TCP and TCP-MAP with blank CT25 (1-10 μM). (D) Represents the data obtained from the encapsulation of Lys(N_3)-MAP and co-encapsulation of TCP and Lys(N_3)-MAP in the NLC compared to peptide conjugate. Results are expressed as mean±SD (n = 4). Statistical analysis between the groups was performed using two-way ANOVA with Tukey's multiple comparisons test (* *p* <0.05; ** *p* <0.01; *** *p* <0.001; **** p <0.0001).

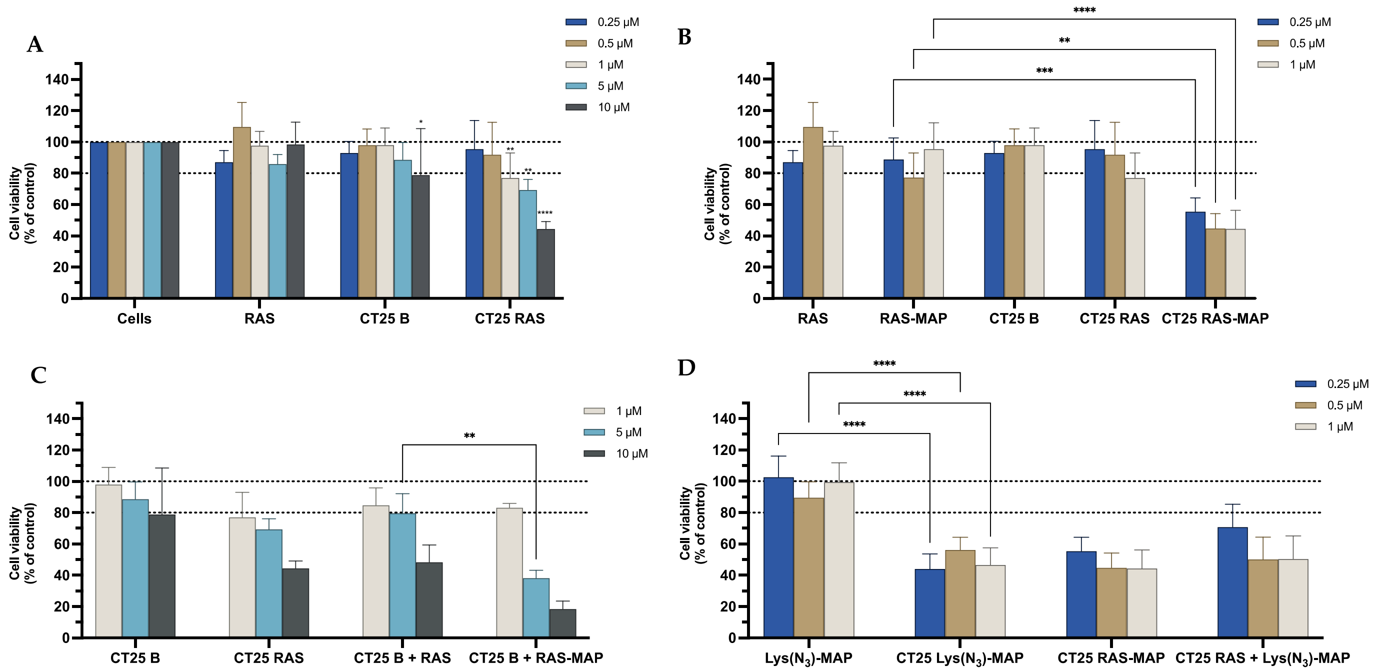

Figure S18 - Relative cell viability of SH-SY5Y cell line measured by the SRB assay after 24 h incubation. (A) represents NLC safety test with the treatment of free RAS, blank CT25 and RAS loaded CT25 (0.25-10 μM). (B) Represents differences in the cell viability of treatment with free RAS, RAS-MAP, blank CT25, RAS loaded CT25, MAP, RAS-MAP loaded CT25 (0.25-10 μM). (C) Represents co-administration of RAS and RAS-MAP with blank CT25 (1-10 μM). (D) Represents the data obtained from the encapsulation of Lys(N_3)-MAP and co-encapsulation of RAS and Lys(N_3)-MAP in the NLC compared to peptide conjugate. Results are expressed as mean±SD (n = 4). Statistical analysis between the groups was performed using two-way ANOVA with Tukey's multiple comparisons test (* p <0.05; ** p <0.01; **** p <0.0001).

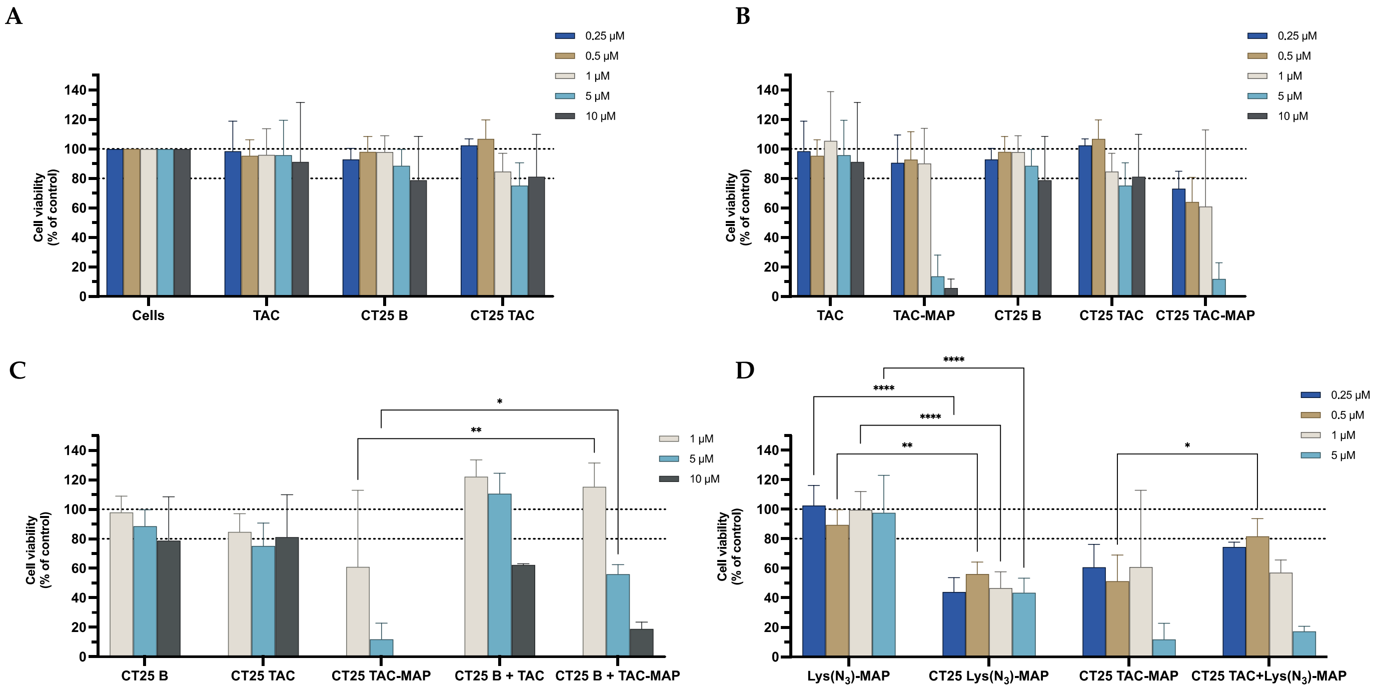

Figure S19 - Relative cell viability of SH-SY5Y cell line measured by the SRB assay after 24 h incubation. (A) represents NLC safety test with the treatment of free TAC, blank CT25 and TAC loaded CT25 (0.25-10 μM). (B) Represents differences in the cell viability of treatment with free TAC, TAC-MAP, blank CT25, TAC loaded CT25, MAP, TAC-MAP loaded CT25 (0.25-10 μM). (C) Represents co-administration of TAC and TAC-MAP with blank CT25 (1-10 μM). (D) Represents the data obtained from the encapsulation of Lys(N_3)-MAP and co-encapsulation of TAC and Lys(N_3)-MAP in the NLC compared to peptide conjugate. Results are expressed as mean±SD (n = 4). Statistical analysis between the groups was performed using two-way ANOVA with Tukey's multiple comparisons test (* p <0.05; ** p <0.01; **** p <0.0001).

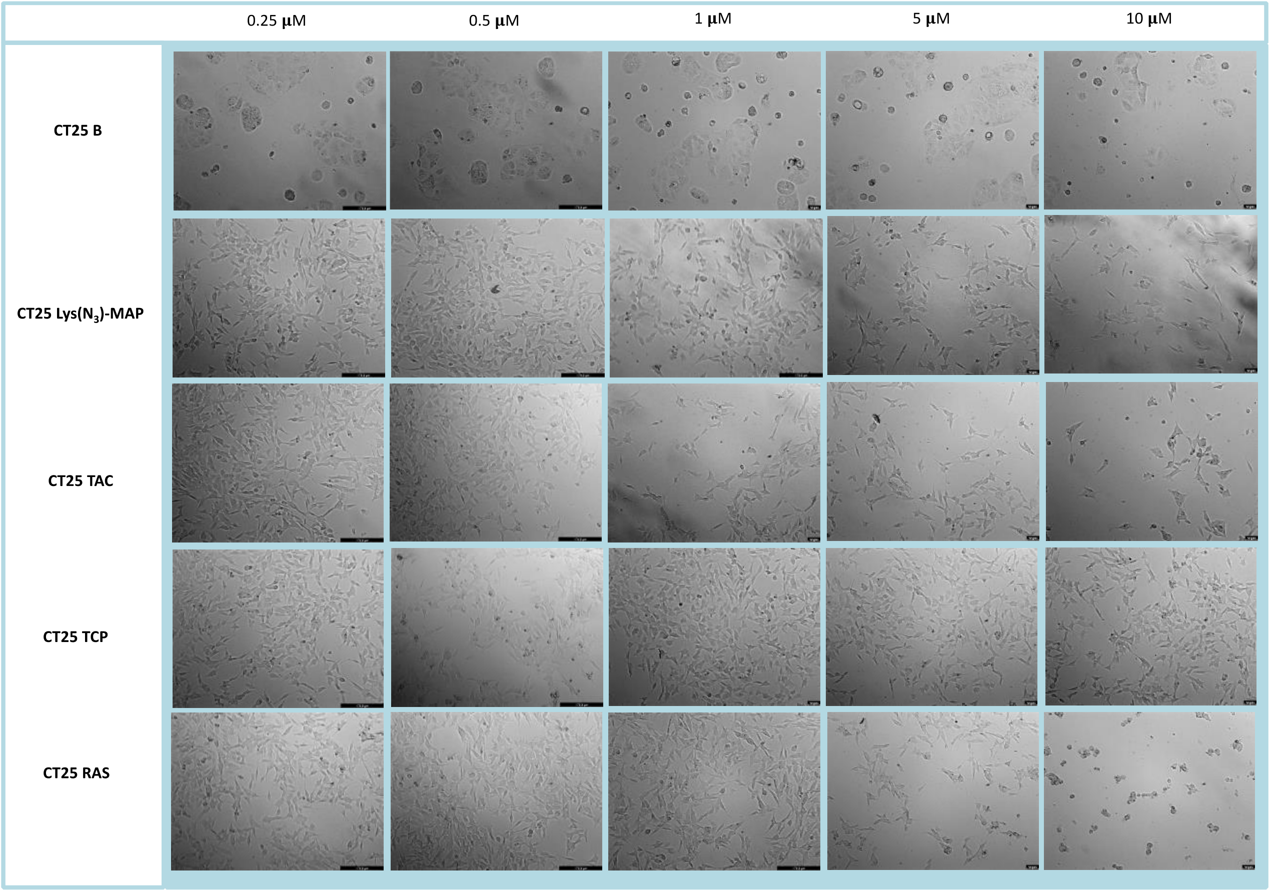

Figure S20 - Microscope cellular visualization through Lionheart FX and Leica DFC350 FX of treatment with free drug and NLC loaded formulations on SH-SY5Y cell line at a concentration of 0.25, 0.5, 1, 5 and 10 μM.

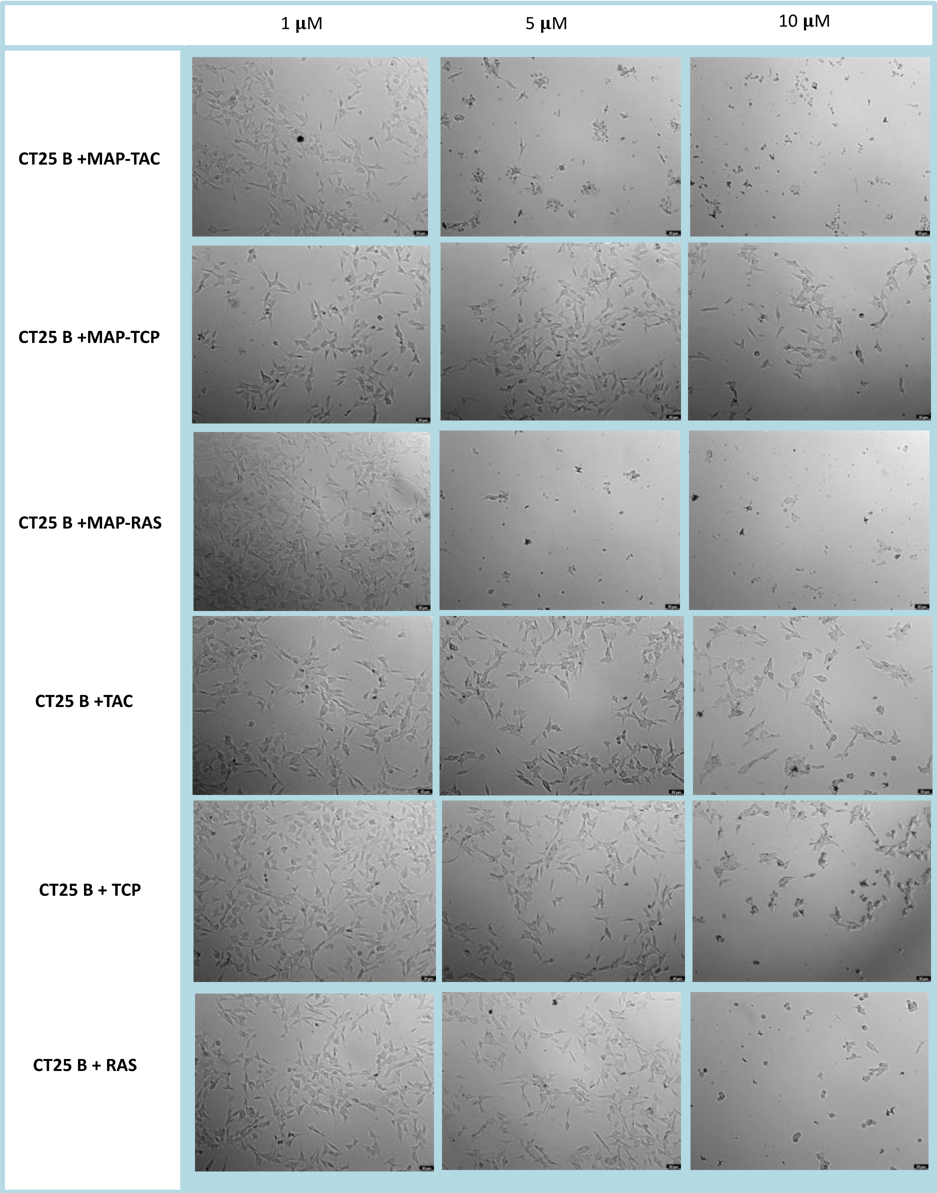

Figure S21 - Microscope cellular visualization through Lionheart FX and Leica DFC350 FX of treatment with free drug and NLC loaded formulations on SH-SY5Y cell line at a concentration of 0.25, 0.5, 1, 5 and 10 µM.
